# High mutation rates limit evolutionary adaptation in *Escherichia coli*

**DOI:** 10.1371/journal.pgen.1007324

**Published:** 2018-04-27

**Authors:** Kathleen Sprouffske, José Aguilar-Rodríguez, Paul Sniegowski, Andreas Wagner

**Affiliations:** 1 Department of Evolutionary Biology and Environmental Studies, University of Zurich, Zurich, Switzerland; 2 The Swiss Institute of Bioinformatics, Lausanne, Switzerland; 3 Department of Biology, University of Pennsylvania, Philadelphia, Pennsylvania, United States of America; 4 The Santa Fe Institute, Santa Fe, New Mexico, United States of America; Université Paris Descartes, INSERM U1001, FRANCE

## Abstract

Mutation is fundamental to evolution, because it generates the genetic variation on which selection can act. In nature, genetic changes often increase the mutation rate in systems that range from viruses and bacteria to human tumors. Such an increase promotes the accumulation of frequent deleterious or neutral alleles, but it can also increase the chances that a population acquires rare beneficial alleles. Here, we study how up to 100-fold increases in *Escherichia coli’s* genomic mutation rate affect adaptive evolution. To do so, we evolved multiple replicate populations of asexual *E*. *coli* strains engineered to have four different mutation rates for 3000 generations in the laboratory. We measured the ability of evolved populations to grow in their original environment and in more than 90 novel chemical environments. In addition, we subjected the populations to whole genome population sequencing. Although populations with higher mutation rates accumulated greater genetic diversity, this diversity conveyed benefits only for modestly increased mutation rates, where populations adapted faster and also thrived better than their ancestors in some novel environments. In contrast, some populations at the highest mutation rates showed reduced adaptation during evolution, and failed to thrive in all of the 90 alternative environments. In addition, they experienced a dramatic decrease in mutation rate. Our work demonstrates that the mutation rate changes the global balance between deleterious and beneficial mutational effects on fitness. In contrast to most theoretical models, our experiments suggest that this tipping point already occurs at the modest mutation rates that are found in the wild.

## Author summary

Mutation is of central importance in biology. It creates genetic variation, the raw material of evolution by natural selection, It can improve traits and organisms, but can also lead to phenomena like cancerous cells and antibiotic resistant pathogens. Increasing the mutation rate can accelerate evolutionary adaptation, even over many thousands of generations in a constant environment. Our study describes the laboratory evolution of asexual *Escherichia coli* strains with a range of mutation rates at levels found in the wild (from wild type to strong mutator). Unexpectedly, evolutionary adaptation was most limited in the populations with the highest mutation rate. Our work suggests that deleterious mutations can begin to limit adaptation at lower mutation rates than previously thought.

## Introduction

Mutation is fundamental to evolution. Without it, evolution cannot occur, because mutation provides the genetic variation necessary for selection and genetic drift. Each new mutation in an individual can increase its fitness, decrease its fitness, or have no effect on its fitness. Unfortunately, most mutations with fitness effects are deleterious, and fitness-increasing beneficial mutations constitute only a small fraction of all possible mutations [1]. The mutation rate can itself evolve, because it is subject to genetic change in the "mutation rate genome", the part of a genome encoding DNA replication and repair systems [2,3]. Here, we characterize the long-term effects of a range of mutation rates on adaptation, as well as the evolution of the mutation rate itself, by evolving multiple replicate populations of asexual *Escherichia coli* in a minimal medium in the laboratory.

Evolutionary adaptation under increased mutation pressure in large non-recombining populations like ours has been explored in past work (all mutations that occur in our *E*. *coli* laboratory strain's genome are linked). The joint effects of mutation and linkage on selection (and the related topics of diversity and the evolution of sex) have been much studied since Fisher [4] and Muller [5] ([6–10], recently reviewed in [11–14]). Under increased mutation pressure, multiple clones within a population may acquire new mutations, and then compete with each other for fixation. While relevant studies show that the speed of adaptation can increase with the genomic mutation rate [10,15–18], they leave open the possibility that extremely high mutation rates could hinder adaptation. This possibility is raised by a variety of models that predict declining fitness in populations with extreme mutation rates. An early, influential, but simple model predicted that a population's fitness will decrease when the rate of mutation increases beyond a critical “error threshold” [19] whose value depends on model details. Other models of populations evolving at high mutation rates are more realistic and take into account phenomena like beneficial mutations and demography. However, they also predict that adaptation can be slowed and eventually reversed at sufficiently high mutation rates by the effects of deleterious mutations [20–24].

Many studies have documented the evolution of increased mutation rates [25–31], which can evolve in certain conditions. For example, after a recent environmental change that creates opportunities for novel adaptations and new beneficial mutations [32,33], a cell with a mutator allele is more likely to produce large-effect beneficial mutations than a cell with a wild-type mutation rate. Because of their improved fitness, cell lineages with newly acquired beneficial alleles (and their linked mutator alleles) can increase in frequency in the population. Thus, hypermutation can readily evolve when mutator alleles hitchhike to fixation with beneficial mutations [34–37].

In the long term, however, hypermutation can be detrimental, because most non-neutral mutations have deleterious consequences [1]. Thus, an individual with a higher mutation rate may accumulate more deleterious mutations overall, which can result in lower fitness. For this reason, selection has been predicted to reduce mutation rates [38]. However, there are several potential reasons why mutation rates may not decline all the way to zero. One of them is that the physiological mechanisms required to improve replication fidelity and DNA repair carry a fitness cost [39–42]. Another is that the power of selection to reduce the mutation rate is limited by population size via the so-called drift-barrier [43,44]. Experimental observations of evolved reductions in the mutation rate have been reported, but are relatively infrequent [27,31,45–50] (reviewed in [51]).

While some previous experiments explored the adaptive responses and mutation rate changes that can take place under increased mutational pressure [46–48,50], they focused on one or two mutation rates, and did not include genomic analyses (except [50]). Here, we sought to provide a uniquely comprehensive empirical data set across a range of mutation rates, including whole genome population sequencing data, mutation rate data, and fitness measurements in a number of environments. To do so, we engineered four isogenic *E*. *coli* K12 MG1655 derivative strains with increased mutation rates and evolved eight replicate populations of each strain for 3000 generations in a serial-transfer experiment. Genomic mutation rates differed more than a hundred-fold among these strains and ranged from *U* = 0.00034 to *U* = 0.036 point mutations per genome per generation by one method of estimation. During evolution, we periodically characterized the growth rate and stationary population density of each population. We also assayed the fitness of evolved populations in a variety of stressful environments. High-throughput population sequencing allowed us to characterize how far our populations spread through sequence space, and to study the mutations occurring in each population.

## Results

**Fig 1:**
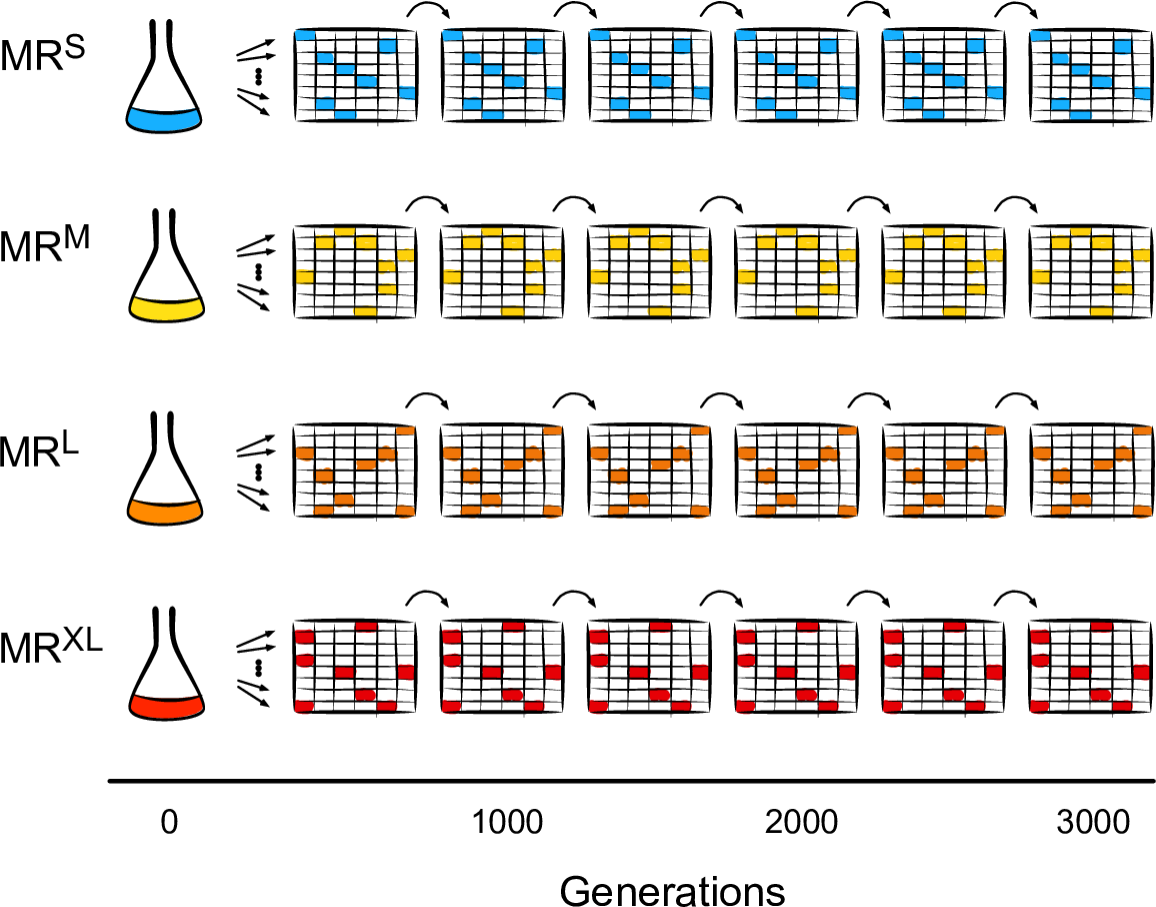
Experimental design. We evolved eight replicate populations for each of four *E*. *coli* strains with increasing mutation rates for nearly 3000 generations, and sequenced all evolving populations approximately every 1000 generations. All eight replicate populations with the same mutation rate shared a single, common ancestor.

The experimental design is summarized in Fig 1. We evolved eight independent replicate populations for each of four isogenic *E*. *coli* strains with increasing mutation rates, and did so for 175 days (about 3000 generations) in minimal medium. The mutation rates, as estimated by mutation to rifampicin resistance, differed 139-fold between the ancestor strains with the lowest and highest mutation rates. We called these strains MR^S^, MR^M^, MR^L^, and MR^XL^ for strains with small, medium, large, and extra-large mutations rates (MR). We evolved all 32 replicate populations at 37°C in 2 mL minimal medium, and diluted them daily 100,000-fold into fresh medium. Because the population density at carrying capacity differed among strains (S1 Fig), the notional effective population size of the evolving replicates also differed, ranging between 42,500 and 92,800 (S2A Fig). Linkage is known to reduce the effective population size [52], so we also estimated its effect on this size (S2B Fig) [53–55]. We found that populations with higher mutation rates generally had lower effective population sizes. We sequenced samples of the evolving populations at generations 0, 1000, 2000, and 3000 to high genome-wide coverage. We measured the mutation rates and tested growth in stressful environments at generations 0 and 3000. We also periodically measured maximum growth rates and growth curves as a proxy for fitness.

### Very high mutation rates can lead to reduced adaptation

**Fig 2:**
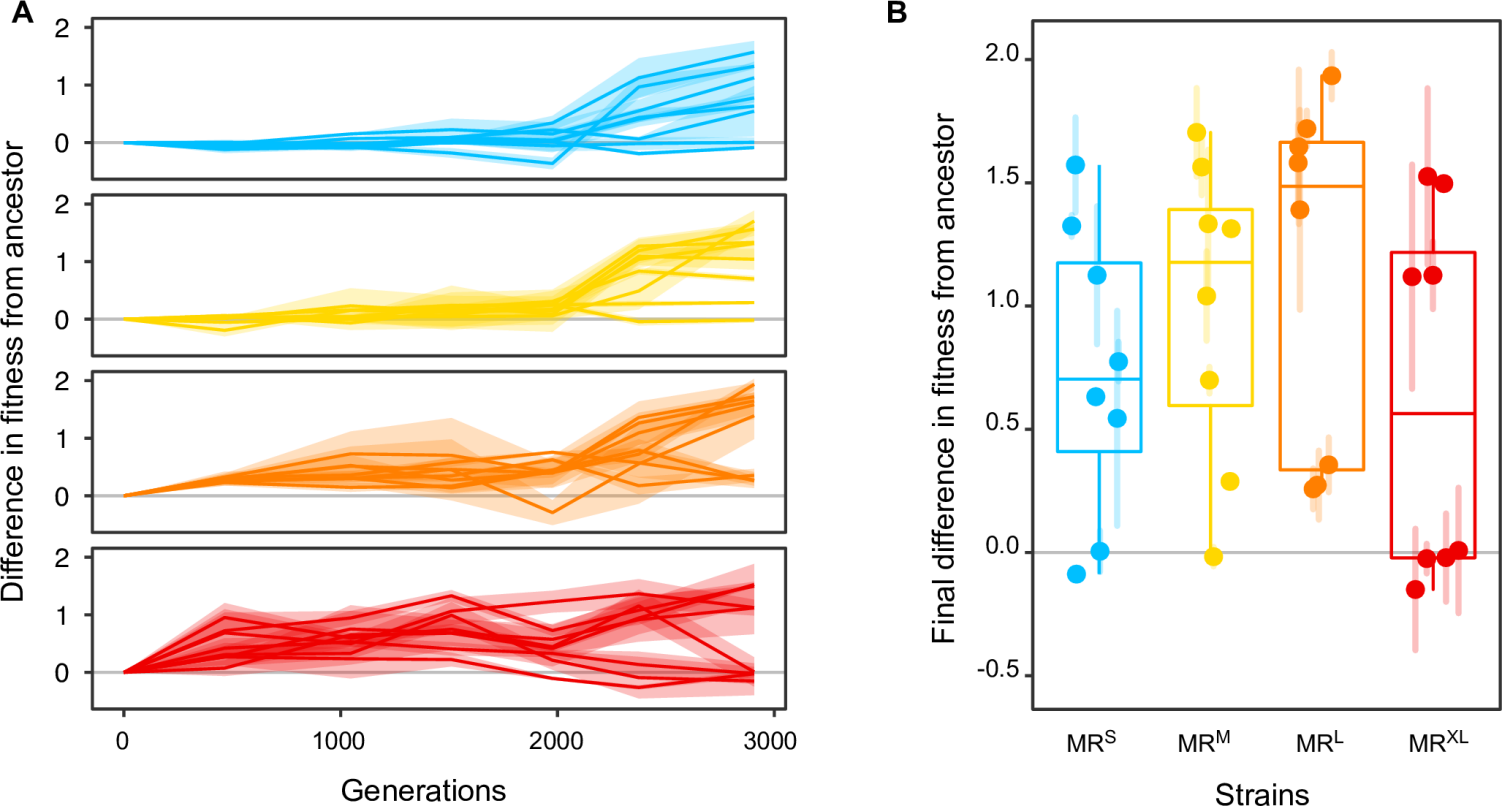
Fitness of the evolving replicate populations relative to their ancestors (A) over time, and (B) at the end of the experiment. A relative fitness value greater than zero indicates that the evolved population has higher fitness than its ancestor. Different colors distinguish data from the MR^S^ (blue), MR^M^ (yellow), MR^L^ (orange), and MR^XL^ (red) strains. Shaded areas (A) or bars (B) indicate s.e.m.

We used a population’s maximum growth rate during exponential growth as a proxy for fitness, and we refer to relative fitness as the difference between an evolved and a reference population, usually the ancestral population. We first measured the fitness of the ancestral strains relative to the *E*. *coli* K12 MG1655 strain and found that the ancestral strains had similar fitness values, with the exception of the MR^L^ strain, which had much lower fitness than the other strains (S3A Fig, linear mixed effects analysis, **Χ**^2^(4) = 248; MR^S^: 0.34±0.02; MR^M^: 0.32±0.02; MR^L^: 0.11±0.02; MR^XL^: 0.25±0.02, relative fitness ± s.e.m., p<2×10^−16^). We next measured the fitness of each evolving replicate population relative to its ancestor at several time points during the experiment (Fig 2, S3B Fig, S4A Fig). At generation 3000, replicate populations with higher mutation rates showed a greater increase in fitness, except for the MR^XL^ strain, which had the smallest fitness increase of all strains (p = 0.002, linear mixed effects analysis, **Χ**^2^(4) = 16; simultaneous tests for general linear hypotheses that relative fitness is unchanged at generation 3000: MR^S^:0.7±0.2, p = 0.004; MR^M^:1.0±0.2, p<4×10^−5^; MR^L^:1.1±0.2, p<1×10^−6^; MR^XL^:0.6±0.2, p = 0.02, evolved fitness difference from ancestor ± s.e.m., significance). In replicate populations with lower mutation rates (MR^S^ and MR^M^) fitness only began to rise substantially after 1000 generations, while in replicate populations with higher mutation rates (MR^L^, MR^XL^) this fitness increase began earlier (Fig 2A; linear mixed effects analysis, **Χ**^2^(4) = 17, p = 0.002; simultaneous tests for general linear hypotheses that relative fitness is unchanged at generation 1000: MR^S^: -0.01±0.07, p = 1.0; MR^M^:0.07±0.07, p = 0.76; MR^L^:0.40±0.07, p<0.0001; MR^XL^:0.57±0.07, p<0.0001, evolved fitness difference from ancestor ± s.e.m., significance). A second growth curve metric that integrates information about the lag phase, growth rate, and carrying capacity yielded similar results (S1 Text). The delays in fitness gains in populations with lower mutation rates are also reflected in reduced fitness variation among replicate populations at any one point in time (S4B Fig). MR^L^ and MR^XL^ populations seem to form two clusters with either high or low (ancestral-like) fitness at the end of the experiment (Fig 2B). In principle, the fitness of populations with lower fitness might either not have increased at all, or it might have increased at first and subsequently decreased again. Examination of individual fitness trajectories (S4A Fig) shows that most MR^XL^ replicate populations with low fitness at generation 3000 gained and then lost fitness again.

### Population sequencing at regular intervals

We sequenced a sample of each heterogeneous evolving population rather than a clone isolated from each population, so that we could estimate the genetic diversity within each sequenced population. We sequenced 100 populations in total: the four ancestor populations with different mutation rates, and eight replicates evolved from each ancestor at generations 1000, 2000, and 3000. Specifically, we sequenced the four ancestor populations after one day of growth just before having split them into their replicate populations. The mean sequence coverage for populations was 364-fold (standard deviation 98), with 99% (83%) of the samples having at least 100-fold (200-fold) coverage across at least 95% of the genome. In virtually all sequenced samples less than 1% of the genome had no sequence coverage (S5 Fig). We identified the frequency of SNPs in each sequenced population and their annotations using breseq, which has been widely used in microbial studies and has been optimized for bacterial data [56] (S6 Fig). We discovered several SNPs at non-zero frequency in these sequenced ancestral populations (1, 3, 2, and 64 loci for MR^S^, MR^M^, MR^L^, and MR^XL^, respectively; S7 Fig), some of which may have been transferred to the evolving populations. Previous studies had suggested that there may be biases in the mutational spectra caused by the reduced efficacy of the *mutL* and *dnaQ* gene products [57–59], but our sequence data shows that any such bias is weak or absent in our strains (S8 Fig).

### Higher mutation rates lead to larger mutant clouds and more high frequency derived alleles

**Fig 3:**
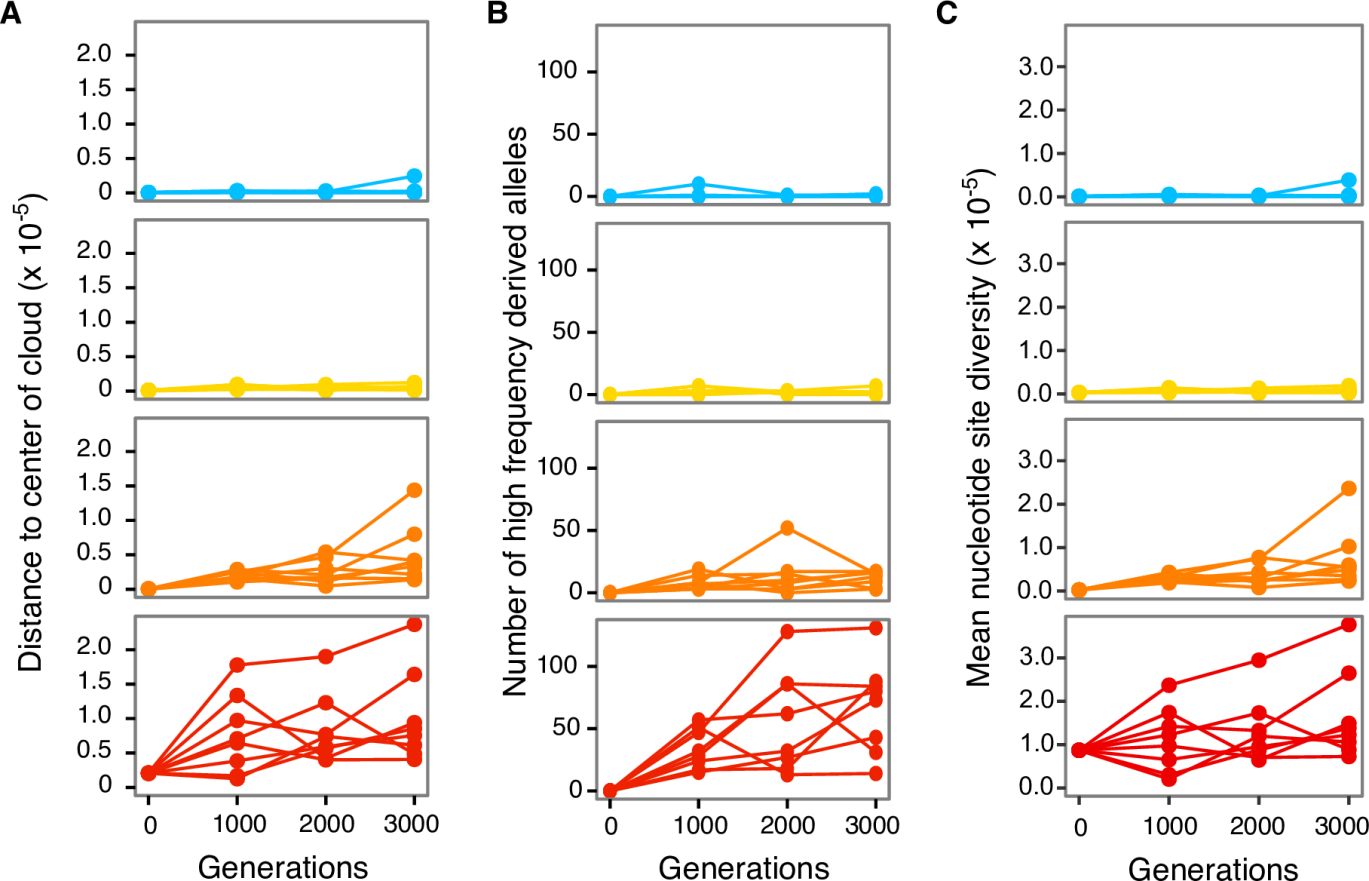
Replicate populations with higher mutation rates have increased genetic diversity and more high frequency derived alleles. Each circle represents (A) the distance of a population to its center (vertical axis), (B) the number of sites with derived alleles at a frequency exceeding 50% (vertical axis), or (C) the mean nucleotide site diversity (vertical axis) in an evolving replicate population over the course of the evolution experiment (horizontal axes). Different colors distinguish data from the MR^S^ (blue), MR^M^ (yellow), MR^L^ (orange), and MR^XL^ (red) strains.

One can view an evolving population as a cloud of mutant individuals in sequence space. We suspected that this mutant cloud would be spread out further in genotype space–indicating greater standing diversity–for populations with a higher mutation rate. To test whether this was indeed the case, we first defined the center of an evolving population as its consensus sequence and then computed the average distance of each population to this consensus. A strain’s mutation rate affected the size of its mutant cloud (linear mixed effects analysis, cube root of diversity taken to ensure homoscedasticy, **Χ**^2^(4) = 79; p = 3×10^−16^; see Methods), such that higher mutation rates led to a larger cloud (Fig 3A; MR^S^:0.005±0.001; MR^M^:0.007±0.001 MR^L^:0.011±0.001; MR^XL^:1.018±0.001, cube root of diversity ± s.e.m.). Similarly, higher mutation rates led to higher levels of mean nucleotide site diversity (Fig 3C).

We also expected evolving replicate populations with higher mutation rates to accumulate more high frequency derived alleles than those with lower mutation rates because populations with more variation are expected to adapt faster. Here, we defined a high frequency derived allele at a given site as a derived allele found in more than 50% of the population. Again, strain identity affected the number of high frequency derived alleles (linear mixed effects analysis, **Χ**^2^(4) = 69, p = 4×10^−14^; see Methods), such that strains with higher mutation rates accumulated more high frequency derived alleles (Fig 3B; MR^S^:0.8±0.3; MR^M^:1.8±0.8; MR^L^:12±2; MR^XL^:68±13, number of high frequency derived alleles ± s.e.m. at generation 3000).

### Beneficial mutations

Most new mutations are thought to be effectively neutral or deleterious, and only a small fraction are beneficial in a given environment [1]. To identify putatively beneficial mutations in our replicate populations, we developed a statistical test that identifies genes in which more replicate populations contain high frequency derived alleles of any one gene than one would expect by chance alone (Methods, S9 Fig). In addition to identifying beneficial mutations, this approach can also identify artifacts such as mutational hotspots or the violation of independence across samples. Our test identified 20 genes with putatively beneficial mutations. Statistical analysis places the highest confidence for the existence of beneficial mutations in eight of these genes (*clpB*: 6/32 populations, p<6×10^−6^, *cspC*: 3/32 populations, p = 3×10^−5^, *mreB*: 5/32 populations, p<6×10^−6^, *pykF*: 16/32 populations, p<6×10^−6^, *rnb*: 7/32 populations, p<6×10^−6^, *rpoC*: 6/32 populations, p<6×10^−6^, *topA*: 9/32 populations, p<6×10^−6^, and *ygeN*: 4/32 populations, p = 4×10^−5^ (S9A Fig, S10 Fig). Most of the mutations are nonsynonymous or nonsense mutations, and are thus likely to affect gene function (S9A Fig). Furthermore, between 70 and 100% of the observed mutations in any one gene occurred at different sites in different replicate populations (*clpB*: 100%, *cspC*: 100%, *mreB*: 91%, *pykF*: 95%, *rnb*: 78%, *rpoC*: 100%, *topA*: 70%, and *ygeN*: 100%), indicating that they occurred *de novo* and independently from each other. The two most commonly mutated genes were *pykF* and *topA*, which encode pyruvate kinase and topoisomerase A, respectively. Pyruvate kinase is a key enzyme in glycolysis, and topoisomerase A can affect the superhelicity of DNA. Both genes have repeatedly acquired beneficial mutations in previous experiments with *E*. *coli* B in glucose minimal medium [60–65]. Similarly, mutations in *cspC*, a stress protein, confer a fitness advantage for *E*. *coli* populations evolving at 37°C and higher [62,66]. Finally, mutations in the RNA polymerase gene *rpoC* and the cytoskeletal gene *mreB* have also been commonly found in laboratory evolution [62,67,68].

Surprisingly, multiple MR^XL^ replicates showed the same nucleotide change in 12 of the 20 putatively beneficial genes. As previously discussed, some mutations arose in the ancestor MR^XL^ population before we split it into its replicate populations (S7 Fig). Specifically, mutations in 10 of the 12 genes with the same nucleotide change across the MR^XL^ replicates were also found in the ancestral population (at a frequency between 3% and 22%), before we had split this population into our replicates (S9B Fig). Only two genes (*yfeZ* and *rrlH*) showed no evidence for such identical, pre-existing mutations, although such mutations may have existed below the detection limit of our sequencing coverage. We cannot conclude that these 12 putatively beneficial genes have a beneficial effect in the MR^XL^ populations, because our statistical test relies on the assumption that the mutations occurred and were subject to selection independently. To know with certainty the phenotypic effects of any of these mutations would require additional empirical data from allelic replacement experiments.

### Growth and survival in stressful conditions

Thus far, the only phenotype we studied was population growth in one environment–the glucose minimal medium in which we conducted the entire experiment. To expand our analysis to other environments, we used Biolog Phenotype MicroArrays, which help measure the growth and respiration activity of a bacterial strain in multiple environments ([69], but see [70] for caveats). These microarrays determine the ability of our strains to grow in the presence of 96 stressful compounds that include antibiotics and heavy metals. We exposed our evolving replicate populations to these stressors only after completion of laboratory evolution, i.e., the populations could not have adapted to them during the evolution experiment. We selected two populations at random from the MR^S^, MR^M^, MR^L^, and MR^XL^ replicate populations at the end of evolution. Remarkably, the two selected MR^XL^ replicates failed to grow in every single one of the 96 environments, as did the MR^XL^ ancestor. One possible explanation is that the MR^XL^ strain is inherently more sensitive to novel environments, including the medium used in the assay. The remaining populations grew in 42–60 (43.8%-62.5%) of the environments, depending on the population. In order to identify any link between mutation rate and growth in these 96 environments for the MR^S^, MR^M^, and MR^L^ replicates, we identified the molecules in which an evolved replicate population grew better (and worse) than its ancestor (S11 Fig). All replicate populations were better able to tolerate stressful conditions than their ancestors in some of the tested conditions (between 8% to 30%), which suggests that some (fortuitously) beneficial mutations have occurred. The two MR^L^ replicate populations we tested tolerated stressful conditions better than the MR^S^ and MR^M^ replicate populations. However, the MR^S^ replicate populations were able to tolerate more stressful conditions than the MR^M^ populations. In sum, these analyses establish no simple association between ancestral mutation rate and stress tolerance after evolution.

**Fig 4:**
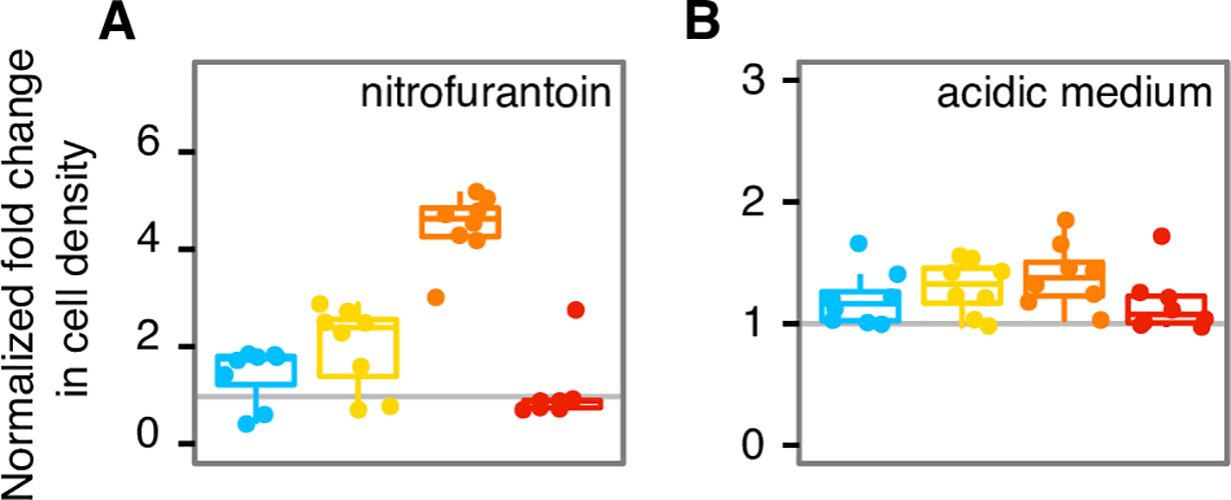
Cell density after 24 hours of growth in stressful conditions increased with increasing mutation rate, except for MR^XL^ replicate populations. We measured the cell density of the MR^S^, MR^M^, MR^L^, and MR^XL^ evolved replicate populations relative to the ancestor (vertical axis) at generation 1000 in (A) medium supplemented with nitrofurantoin (2.2 μg/mL) and in (B) acidic medium (pH 5.25). The MR^S^, MR^M^, MR^L^ replicate populations performed better with increasing mutation rate, except for the MR^XL^ replicate populations which performed worst among all populations. See S12 Fig for data on additional timepoints, and nitrofurantoin and pH conditions. Different colors distinguish data from the MR^S^ (blue), MR^M^ (yellow), MR^L^ (orange), and MR^XL^ (red) strains.

To study the evolutionary dynamics of growth in stressful conditions over the course of the experiment, we periodically tested the growth of the ancestor and all evolving replicate populations in two stressful conditions: the antibiotic nitrofurantoin (a specific, “narrow” stressor) and acidic media (a broader stressor). Nitrofurantoin is a nitrofuran antibiotic with multiple mechanisms of action. Resistance to nitrofurantoin is conferred by mutations in two genes (*nfsA* and *nfsB*), and has a fitness cost in the absence of the antibiotic [71]. Thus, resistance mutations are unlikely to exist at appreciable levels as part of the standing variation in populations not exposed to nitrofurantoin. *E*. *coli* is known to tolerate acidic conditions due to several acid resistance systems, one of which depends on the alternative sigma factor σ^S^, encoded by the *rpoS* gene [72,73]. For both nitrofurantoin-containing and acidic media, we computed the fold change in growth (the cell density after 24 hours) of the evolved populations relative to their ancestors, controlling for changes in carrying capacity (see Methods, Fig 4, S12 Fig). We found that replicate populations with the highest mutation rates grew more slowly in nitrofurantoin than their ancestor. In contrast, populations at low and intermediate mutation rates grew faster than the ancestor (see Methods, p = 5×10^−8^, linear mixed effects analysis, **Χ**^2^(4) = 40; MR^S^:0.85±0.15; MR^M^:0.49±0.15; MR^L^:0.94±0.15; MR^XL^:-0.29±0.15, log fold change ± s.e.m., positive log fold change indicates evolved strains grew better, and negative log fold indicates ancestor strains grew better). The ancestral mutation rate also affected growth in acidic media. In contrast to nitrofurantoin, all strains showed increased growth, but qualitatively similar to nitrofurantoin, the MR^XL^ replicates showed the smallest growth rate increase at low pH (p = 2×10^−8^, linear mixed effects, **Χ**^2^(4) = 42); MR^S^: 1.23±0.09; MR^M^:1.39±0.09; MR^L^:1.56±0.09; MR^XL^:1.15±0.09, fold change ± s.e.m., fold change > 1 indicates evolved strains grew better, and fold change < 1 indicates ancestor strains grew better). In sum, growth in two stressful conditions, nitrofurantoin-containing and acidic media, improved with increasing mutation rates (and thus increasing diversity), except for the MR^XL^ replicates which showed a relative reduction in growth.

### Mutation rate decreased for the highest mutation rate replicates

**Fig 5:**
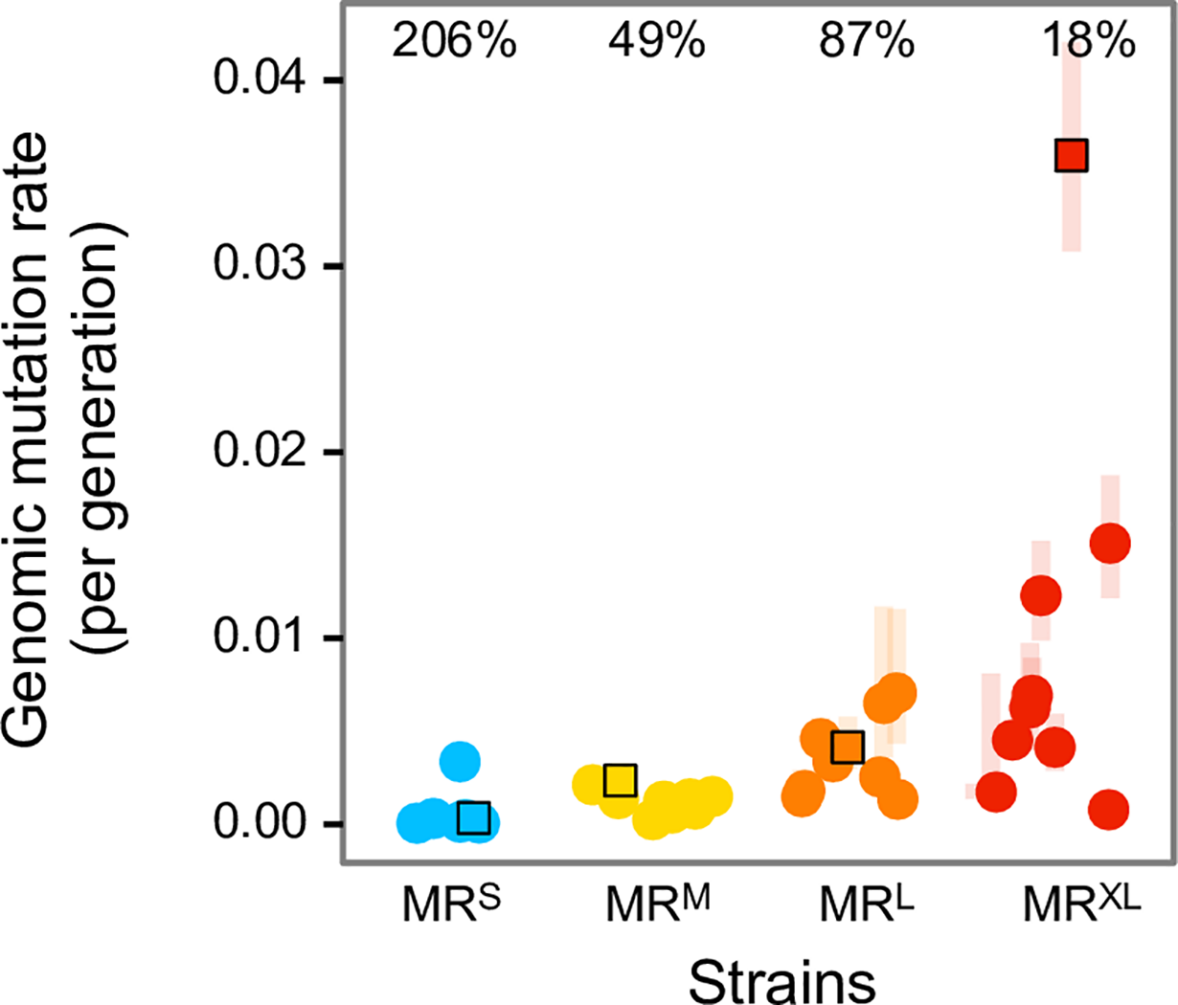
Evolution of the mutation rate. Ancestral genomic mutation rates are shown as squares, evolved mutation rates at generation 3000 as circles, and 95% confidence intervals of mutation rate estimates are shaded. Each evolved strain's mean change in mutation rate is shown as the percentage of its ancestor's mutation rate. Data from MR^S^ strains are shown in blue, from MR^M^ strains in yellow, from MR^L^ strains in orange, and from MR^XL^ strains in red.

We measured the mutation rate of one randomly selected clone from each evolved replicate population at generation 3000, and of the population’s ancestral strain. To this end, we used fluctuation assays for mutations that cause rifampicin resistance, and estimated the genomic mutation rate *U* using Drake's approach [74]. The mutation rates of the evolved MR^XL^ replicates decreased on average by 556%, reaching 18% of the ancestor’s mutation rate (2%-42%, depending on the replicate); Fig 5, S3 Table). At the end of the evolution experiment, the mutation rates in the MR^XL^ populations were no longer statistically distinguishable from those of the MR^L^ replicates (Wilcoxon rank sum test, p = 0.38). The evolved populations’ mutation rates for the MR^M^ and MR^L^ strains also tended to decrease (MR^M^: 49% of the ancestor's mutation rate on average, ranging between 11%-90% of the ancestor; MR^L^: 87%, range 32%-170%). In contrast, the replicates from the MR^S^ strain increased their mutation rate somewhat, to 206% of the ancestor's mutation rate (range 16%-1000%). Having estimated the mutation rates for the ancestor and evolved populations, we also wanted to examine whether prominent theoretical models that predict declines in mean population fitness at high mutation rates apply to our populations (S2 Text). While some of the models we studied (e.g., that of mutational load) predict a small reduction in fitness at the highest mutation rates we employed, none of them could account for the magnitude of the loss of adaptation we found in several of the MR^XL^ replicates (S2 Text; Fig B, Fig C in S2 Text).

Because mutation rates changed between the beginning and the end of the experiment, we wondered whether the final mutation rates were correlated with our measured phenotypes. We found significant correlations between a replicate's mutation rate and its effective population size, standing genetic diversity, and number of high frequency derived alleles, but no correlations between a replicate's mutation rate and its final relative fitness, or normalized cell density after 24 hours of growth in acidic medium or medium containing nitrofurantoin (Spearman's rank correlation, S13 Fig). Interpretation of these results requires caution for two reasons. First, for any one population, we do not know exactly when (during the 3000 generations of evolution) the mutation rate changed from its ancestral value. Second, we compared the mutation rate of a single randomly-selected clone from populations which can have considerable genetic diversity, and thus potentially also show diversity in mutation rates. Despite these caveats, we found that the correlations between a representative clone's mutation rate and our other metrics are consistent with our previous analyses and figures (Fig 4, S2 Fig, S12 Fig), which simply considered the effects of ancestral mutation rate (strain identity).

### Changes in the mutation rate genome

We call the set of genes potentially involved in modulating the mutation rate the "mutation rate genome".

We wondered whether this part of the whole genome was a preferential target of mutation or selection in our experiments. To find out, we first identified a set of 96 genes potentially involved in modulating the mutation rate (S2 Table) from the literature and EcoCyc [49,75–77]. If mutations or selection did not preferentially affect the mutation rate genome, the amount of genetic change we observe in it would be proportional to its length relative to the rest of the genome. This is indeed the case: We counted the number of synonymous mutations occurring at any frequency in any replicate population at generation 3000, and observed no statistically significant increase in the incidence of such genetic change in the mutation rate genome for any of our evolving strains (S14A Fig). We also found no difference in mean diversity between synonymous sites in the mutation rate genome relative to the rest of the genome (S14B Fig).

Although the mutation rate genome is not a preferential target of genetic change, its genes still accumulated many non-synonymous and nonsense changes, which are the kinds of changes that are especially likely to affect protein function (S15 Fig). To identify mutant alleles putatively associated with the decrease in mutation rates we had observed in MR^XL^ replicate population after 3000 generations (Fig 5), we identified nonsynonymous or nonsense mutations in the mutation rate genome with an allele frequency of at least 50% in any MR^XL^ evolved replicate population. Mutations in ten genes met these criteria (*rpoS*, *umuC*, *dinB*, *dinG*, *dps*, *glyS*, *glyW*, *mutL*, *phr*, and *vsr*), and two were found in multiple replicate populations (*rpoS*: 7 of 8; *umuC*: 2 of 8). The *rpoS* gene encodes the alternative sigma factor, σ^S^, which activates the stress response in *E*. *coli* (reviewed in [78]). Populations with *rpoS* mutations can hold a fitness advantage in nutrient-limiting environments [79], but at a cost to fitness in a variety of stressful environments [28,80]. Because σ^S^ is a bacterial transcription factor, it can only affect the mutation rate indirectly, by changing the expression of proteins directly involved in DNA copying, repair, and proofreading. For example, σ^S^ modulates the expression of the error prone DNA Polymerase IV encoded by *dinB* [81]. We found the same *rpoS* N124D mutation in 2.5% of the individuals in the ancestral MR^XL^ population and in all eight evolved replicates. (This mutation reached 40.1% in MR^XL^_1_ and 100% in the rest of the replicates.) Thus, the mutation was likely distributed to the eight replicate populations from the ancestor, and either increased in frequency due to its direct fitness effects, or because it was hitchhiking with a beneficial mutation. The MR^XL^_4_ and MR^XL^_6_ replicate populations each acquired different non-synonymous mutations in *umuC*, which encodes DNA polymerase V. Each of the remaining genes with high frequency mutant alleles in a single replicate population were involved in DNA repair and replication (*dinB*, *dinG*, *glyS*, *glyW*, *mutL*, *phr*, *vsr*) or protection of DNA in stationary phase (*dps*) in a single replicate population and could have also affected the evolved mutation rate.

## Discussion

Here, we studied the effects of mutational pressure on evolutionary adaptation and the evolution of the mutation rate itself. To this end, we engineered four isogenic *E*. *coli* K12 MG1655 derivative strains with increasing mutation rates (MR^S^, MR^M^, MR^L^, and MR^XL^), and evolved them for 3000 generations. Our smallest (wild-type) ancestral mutation rate (MR^S^: U = 0.00034 per genome per generation) was somewhat smaller than rates estimated in *E*. *coli* strains using a similar experimental approach (U = 0.0025) [39,74], but similar to those estimated in a wildtype *E*. *coli* B strain using a sequencing approach (U = 0.00041) [82]. At the opposite extreme was our strain with the highest ancestral mutation rate (MR^XL^). We originally expected this strain to have a mutation rate approximately 4500-fold higher than wildtype [35], consistent with the large effects that mutations in the *dnaQ* and *mutL* genes have on the mutation rate [75]. However, our measurements of this rate demonstrated that it was lower than expected (MR^XL^: 139-fold higher than our wildtype; U = 0.036 per genome per generation). The discrepancy could in principle be due to the acquisition of an anti-mutator allele during the transfer of the strain between laboratory locations. Alternatively, our mutation rate could be an underestimate for technical reasons discussed in the Methods. The mutation rate for our MR^XL^ strain was also somewhat lower than that of a hypermutable clone which spontaneously evolved from an *E*. *coli* B strain [50] (*mutT*: U = 0.061). The mutation rate of our hypermutable MR^XL^ strain is low enough that we expected its populations to be viable [21]. In sum, we conducted our experiments with strains having a range of viable mutation rates, from wildtype (MR^S^), to a 16-, 22-, and 139-fold higher mutation rate (MR^M^, MR^L^, and MR^XL^).

We first characterized the general patterns of adaptation in our four strains, and found that their fitness increased significantly by generation 3000 for all replicate populations. Previous experimental evolution studies in constant environments have observed fitness gains that are initially large but decrease over time [17,18,83,84], which is consistent with diminishing returns epistasis, in which the size of the fitness gain in an evolving population depends on its current fitness, such that populations with lower fitness can improve their fitness to a greater extent [85,86]. However, our fitness trajectories differ from those predicted by diminishing returns epistasis in two ways. First, they do not show a decreasing fitness gain over time [18]. Second, the mean fitness of replicate populations with small or modestly high mutation rates (MR^S^, MR^M^) did not immediately improve, but unexpectedly remained largely unchanged for the first 1000 generations (compared to [87]). While delayed adaptive response is consistent with a lower overall beneficial mutation supply rate, it may not be sufficient to explain our observations. We expected to wait just 44 generations for a new beneficial mutation to establish in our slowest-evolving replicate population (S3 Text). It also raises the possibility that even at moderately high mutation rates, contingent evolution [88], in which the timing and the order of mutational events affects a population’s adaptive evolution, may be important in our populations. An instance of such contingent evolution has been documented in *E*. *coli* [89,90], but the higher mutation rate of some of the strains used in our evolution experiment makes contingent evolution a less likely explanation for delayed adaptation.

We next characterized the effect of mutational pressure on adaptation. We found that strains with higher ancestral mutation rates increased in fitness more than those with lower mutation rates, except for MR^XL^ populations, which we will discuss below. These observations are in agreement with theory [15,91] and previous experimental studies which found that large asexual populations of *E*. *coli* [17,50] and yeast [15] with high mutation rates outperformed those with low mutation rates. If we just consider relative fitness after 1000 generations, our data from our four strains are consistent with expectations: MR^S^ and MR^M^ populations have lower mean relative fitness than MR^L^ and MR^XL^. It is only thereafter that the fitness of MR^XL^ populations stops increasing, such that they have lower mean fitness at generation 3000 than the MR^S^, MR^M^, and MR^L^ strains. We do not actually observe the loss of fitness on average across the MR^XL^ replicate populations, but rather a prolonged period in which fitness remains unchanged as a whole. Interestingly, however, the fitness of several MR^XL^ replicate populations decreases from its maximum and arrives at a value that is approximately equal to that of the ancestral population. This is reminiscent of models of extreme mutational pressure developed over the past forty years that predict reduced adaptation and eventual extinction [19,20,22,92–94]. However, these models predict a loss of fitness only at higher mutation rates than we observed, and require unrealistic assumptions (S2 Text), together emphasizing the importance of additional theoretical work. Another possibility is Hill-Robertson interference [7], which can reduce the rate of adaptive evolution by background selection—negative selection against deleterious alleles that removes the most deleterious lineages from a population—and can reduce genetic diversity [8,12]. Empirical evidence supports the action of this mechanism in natural populations of several eukaryotic species (reviewed in [13,14]). However, because background selection removes deleterious mutations from a population, it cannot alone reduce the fitness of a population and it can therefore not explain the loss of fitness we observed in the three MR^XL^ replicates. Overall, our observations support the notion that reduced adaptation can manifest itself at smaller mutation rates than previously thought (*U = 0*.*036* in the MR^XL^ strain), even though more than 1000 generations may be needed to manifest its effects.

This observation is all the more striking, because the mutation rate itself had decreased dramatically for all MR^XL^ populations after 3000 generations (and much less so in the MR^S^, MR^M^, MR^L^ populations). While a lowering of the mutation rate has been previously observed [46–48,50] and predicted to be favored in some conditions [38,40,42,43,95], its extent and consistency across multiple of our evolving populations is remarkable. The mutation rate decrease probably did not occur very early during evolution, because the MR^XL^ populations show greater genetic diversity than all other populations throughout the experiment (Fig 3). The decreasing mutation rate, together with the observation that the MR^XL^ populations failed to adapt after more than 1000 generations, suggests that the maladaptive effects of hypermutation begin at even lower mutation rates than those in our initial MR^XL^ strain. While we cannot predict whether our hypermutable populations would eventually go extinct, the observation that their mutation rate can decrease adaptively makes this less likely. Indeed, recent mutation accumulation experiments with small bacterial populations suggested that populations with higher mutation rates tend to go extinct more often and have reduced fitness than populations with lower mutation rates [47]. Of the several "mutation rate genome" genes mutated in MR^XL^ strains, only *rpoS* was found in all eight evolved MR^XL^ replicate populations. *rpoS* encodes for the stress response modulator σ^S^ that can indirectly affect the mutation rate through transcriptional changes. However, we cannot definitively identify the proximal mechanisms driving the drop in mutation rates using bioinformatics alone. Future experimental studies to evaluate the effect of each "mutate rate genome" mutant allele on the mutation rate and fitness would be necessary.

We emphasize that all our experiments use asexual populations, and that the evolutionary dynamics of mutation rates and adaptation may be different in sexual, recombining populations. For example, in our non-recombining populations, any mutator allele remains completely linked to the (mostly deleterious) mutations it helps bring forth, resulting in indirect negative selection on the mutator allele. However, such an allele and its associated mutations can become unlinked in recombining populations, which reduces the strength of indirect selection on the mutator allele (see [33,39] for reviews). Additionally, beneficial and deleterious alleles can become unlinked in recombining populations, which can lead to increased levels of adaptation and diversity (see [13,14] for reviews).

We also characterized the effect of mutational pressure on the ability of an evolving population to grow better (or worse) than its ancestor in a variety of chemically novel environments, which contain chemical agents that include heavy metal stressors, antibiotics, or acids. Importantly, our populations were never exposed to any of these conditions during the evolution experiment. *A priori*, we reasoned that two outcomes were possible. First, populations with high mutation rates may grow better in novel environments, because they can accumulate more beneficial mutations while evolving in their original environment, and these mutations may also be beneficial in novel environments through pleiotropy. High mutation rate populations can also generate more genotypic diversity, which in turn increases the chances that a population harbors a clone with a latent beneficial mutation that allows it to grow better in a novel environment. Such latent beneficial mutations can indeed occur, as demonstrated by the classic fluctuation test, which relies on such mutations to estimate mutation rates towards resistance to lethal selection [96,97]. Second, populations with high mutation rates may grow worse in novel environments, because they may accumulate more mutations that are either beneficial or neutral in the current environment, but deleterious in a novel environment. Such latent deleterious mutations do indeed exist [36,70,98]. In sum, strains with high mutational pressure may harbor more latent beneficial alleles, but also more latent deleterious alleles, and it is not clear *a priori* which dominates in their effect on fitness.

We conducted two tests on how mutational pressure can affect growth in novel conditions. In the first, we measured the growth of eight evolved replicate populations (two each from MR^S^, MR^M^, MR^L^, and MR^XL^) in 96 chemically novel environments. This test did not yield a clear association between mutation rate and growth for our MR^S^, MR^M^, and MR^L^ populations. However, it yielded a very clear pattern for our MR^XL^ populations: They were not able to grow in any one of these environments, which illustrates that at the highest mutation rates we consider, latent deleterious mutations outweigh beneficial ones in both the ancestor and evolved populations. One possible explanation is that the MR^XL^ strain is inherently more sensitive to novel environments, including the assay environment. Because the MR^XL^ ancestor population could not grow at all, we were unable to further quantify the effect of the highest mutation rate in these 96 novel environments. In the second test, we periodically measured growth of all 32 replicate populations (relative to their ancestors) in two stressful conditions: the antibiotic nitrofurantoin (a specific narrow stressor) and an acidic medium (a broader stressor). For both, we found that strains with higher ancestral mutation rates could grow better than those with lower mutation rates, except for MR^XL^ replicate populations, which grew worst of all populations. This experiment shows that latent beneficial alleles may predominate at low and intermediate mutational pressure, but no longer at high mutational pressure. Our observations are consistent with a previous study showing that multidrug resistance in *E*. *coli* is favored by intermediate mutation rates [99].

In sum, a modest increase in mutation rates can provide an evolutionary advantage in both the constant environment of our long-term laboratory evolution experiment and in novel environments. This advantage disappears at the highest mutation rates (U = 0.036) we considered, where populations show signs of decaying adaptation and poor performance in novel environments. These mutation rates are below those commonly considered to limit adaptation, and highlight the need for additional theoretical work. Our observations show that biological systems may be more sensitive to mutational pressure than simple theoretical models suggest, at least when the effects of mutations are allowed to accumulate over many generations. This observation may improve the prospects of using elevated mutagenesis to drive pathogen or tumor populations to extinction [20,100–104], if high mutation rates can be sustained for a sufficiently long amount of time.

## Methods

### Bacterial strains

We utilized four isogenic *E*. *coli* strains derived from K12 MG1655 that have increasing mutation rates. We refer to these strains as the MR^S^, MR^M^, MR^L^, and MR^XL^ strains, corresponding to small (S), medium (M), large (L), and extra-large (XL) mutation rates. Strain genotypes are summarized in Table 1. We obtained MR^M^ and MR^XL^ strains from our previous experiments (therein called the single- and double-mutator, respectively) [35]. MR^M^ has a non-synonymous (A120T) mutation in the *mutL* gene relative to the *E*. *coli* wild type. This gene is involved in the methyl-directed mismatch repair system. We previously constructed the MR^XL^ strain by P1 transduction of the *dnaQ* gene from the *E*. *coli* CSH116 strain, which has a non-synonymous mutation, T15I, in the *dnaQ* gene, into the MR^M^ strain [35]. The *dnaQ* gene encodes the epsilon subunit of DNA Polymerase III; mutations in this gene can disrupt proofreading. We constructed the mutator strain MR^L^ by replacing the *mutL* region in MR^M^ with the *mutL* region from ES4 with a kanamycin resistance gene inserted upstream of the region, using the method of Datsenko and Wanner [105]. We then excised the kanamycin resistance gene using pCP20 [106], which left a small scar immediately upstream of the *mutL* gene. We constructed the low mutation rate strain MR^S^ by using P1 transduction to replace the error-prone *mutL* region in MR^M^ with the wildtype allele from CAG12073 [107]. We confirmed the mutation rates of these ancestral strains using fluctuation tests [108] (see "Mutation rate measurements and calculations" for details), and found that the MR^M^, MR^L^, and MR^XL^ strains had 16-, 22-, and 139-fold higher mutation rates to rifampicin resistance than MR^S^. We also confirmed our manipulations of the MR^S^, MR^M^, MR^L^, and MR^XL^ strains by sequencing their genomes (S1 Table). In additional to these strains, we used the strain *E*. *coli* K12 MG1655, which we obtained from Yale's Coli Genetic Stock Center.

**Table 1:** Strains and plasmids used in this work.

Strain	Description	Details	Source
MR^S^	*mutL*, *dnaQ* wildtype	*F-, fhuA2, lacY1, tsx-1 or tsx-70, glnV44 (AS), gal-6, λ-, xyl-7, mtlA2, cycA30::Tn10*	This experiment
MR^M^	Impaired mismatch repair (*mutL*)	*F-, fhuA2, lacY1, tsx-1 or tsx-70, glnV44 (AS), gal-6, λ-, xyl-7, mtlA2, mutL13* (Yale's Coli Genetic Stock # 4816)	[35]
MR^L^	*mutL*, *dnaQ* wildtype	*F-, fhuA2, lacY1, tsx-1 or tsx-70, glnV44 (AS), gal-6, λ-, xyl-7, mtlA2*	This experiment
MR^XL^	Impaired mismatch repair (*mutL*), impaired proofreading (*dnaQ*)	F-, fhuA2, lacY1, tsx-1 or tsx-70, glnV44 (AS), gal-6, λ-, xyl-7, mtlA2, mutL13, yafC502::Tn10, dnaQ905	[35]
ES4	Source for wildtype mismatch repair gene (*mutL*) in MR^L^	*F-, fhuA2, lacY1, tsx-1 or tsx-70, glnV44 (AS), gal-6, λ-, xyl-7, mtlA2, purA45*	[109]
*E*. *coli* K12 MG1655	Previously sequenced as the *E*. *coli* K12 reference genome	*F-, LAM-, rph-1* (Yale's Coli Genetic Stock # 7740)	[110]
CAG12073	Wildtype mismatch repair (*mutL*) linked to tetracycline resistance	*λ-, rph-1, cycA30::Tn10*	[107]
pKD4	Plasmid containing *bla*, *FRT*, and *kan*		[105]
pKD46	Plasmid containing *bla* and *araBp-gam-bet-exo*		[105]
pCP20	Plasmid containing *FLP* and *cat*		[106]

### Evolution experiment

See Fig 1 for an overview. We evolved eight independent replicates from populations starting from a single clone for each of the MR^S^, MR^M^, MR^L^, and MR^XL^ strains for 175 days (2907 generations) in 48-well plates (Fluka 15758-500G-F) in 2 mL of Davis Minimal broth [111] supplemented with 1000 mg/L glucose (‘DM1000’) at 37°C with shaking at 400 rpm in a microtiter plate shaker (Stuart Microtiter 51505).

Each plate held 24 populations arranged in a checkerboard pattern, such that each well was surrounded only by wells with blank medium, and the populations were assigned to the 24 wells at random by a custom R script. We diluted each culture 100,000-fold every 24 hours into fresh DM1000 medium, which allows almost 17 generations of growth per day. Every 7 days, we archived each evolving population by adding 400 μL of 50% glycerol to 800 μL of stationary phase culture and freezing at -76°C, estimated the cell density by plating subsamples onto LB (Difco 244620) plates with 1.5% agar (Sigma A1296-1KG), and froze cell pellets from 800 μL of stationary phase culture for eventual genome sequencing. We delayed the start of the MR^S^ replicates by 63 days for technical reasons.

We controlled for contamination in several ways. First, if we observed growth in an empty well, we repeated the transfer from the previous day's 48 well plate stored at 4°C. Second, we periodically checked each evolving culture for contamination by confirming its resistance profile and approximate mutation rate using spot tests. In short, we spotted 25 μL from each evolving culture onto tetracycline (10 μg / mL) and rifampicin (100 μg / mL) plates, and incubated overnight at 37°C. MR^S^ and MR^XL^ replicates can grow on tetracycline, and the replicates with higher mutation rates display more colonies on rifampicin. Cross-contamination occurred once, which prompted us to restart the experiment from the most recent set of uncontaminated glycerol stocks (day 98 for MR^M^, MR^L^, and MR^XL^ replicates; day 35 for MR^S^ replicates). Third, we examined the genome sequence data for cross-contamination, but detected no evidence for cross-contamination in it.

### Effective population size

For populations that do not have a constant number of cells, the effective population size is given by the harmonic mean of population sizes over the course of the dilution and growth cycles of the experiment. Previous studies have estimated the effective population size only from the size of the bottleneck measured during one dilution [112,113]. In contrast, because we recorded the census size of the population at carrying capacity (*N*_*max*_) every 7 days, we were able to estimate the effective population size as the harmonic mean of the population sizes both at the beginning and at the end of a cycle of growth and dilution. To obtain *N*_*max*,*d*_ at any one day *d*, we counted the number of cells in each evolving replicate population in stationary phase just before transferring the population into fresh media. We did so by plating serial dilutions in duplicate on LB agar plates and incubating overnight at 37°C. We discarded plates with fewer than 20 or more than 700 colonies for the purpose of this analysis. Because at the end of each growth cycle we diluted our cultures 100,000-fold into fresh medium, a total of *G* = log_*2z*_(10^5^) = 16.61 cell generations (floor(log_*2*_(10^5^)) ≈16 complete cell generations) elapsed during each growth cycle, and the minimum population size was *N*_*min*,*d*_ *= N*_*max*,*d*_ × 10^−5^. Thus, during each generation *g* of each growth cycle, a population assumed population sizes

$$N_{d,g}=\left\{ \begin{array}{cc} 2^{g}N_{min,d}, & \mathrm{if}\;0\leq g\leq 16\\ N_{max,d}, & \mathrm{if}\;g=17 \end{array}\right..$$

(Because the precise number of generations in each dilution cycle is log_*2*_(10^5^) = 16.61, we included the final number of cells *N*_*d*,*17*_ *= N*_*max*,*d*_ in this calculation). We then determined the nominal effective population size (*N*_*e*_) of a replicate population during its entire lab evolution as

$$N_{e}=25\times 18/\sum_{d=1}^{25}\sum_{g=0}^{18}\frac{1}{N_{g,d}}$$

which is the harmonic mean of all the population sizes. We calculated it for all 25 days on which we collected population size data. The number 18 corresponds to the total number of generations *g* for which we computed population sizes during any one of these 25 days. We also estimated the effect of linkage on reducing the effective population size due to background selection or interference selection [53–55,114]. To this end, we used the R functions (GordoNe and GoodNe, respectively, available from [53]), where we take the size of the deleterious selective effect as *s* = 0.03 [115] and use *U* as an upper bound on *U*_*d*_ to obtain rough estimates of linkage's effect on effective population size.

### Fitness measurements

We periodically obtained a proxy for the fitness of the evolving strains by measuring growth curves of the archived populations. For each time point, we restarted all evolving populations as well as three replicates from each ancestral population and three replicates of wild type *E*. *coli* K12 MG1655 from glycerol stocks in 2 mL of DM1000 and incubated them overnight at 37°C with shaking. We then diluted the overnight cultures 50-fold into 200 μL final volume of DM1000 in 96-well plates (TPP 92096), and incubated them in a plate reader (Tecan Infinite Pro F200) for 18 hours at 37°C with shaking. During this time, we read the absorbance at 600 nm every 10 minutes. We fit the classic logistic equation describing population growth to the data [116], using the Growthcurver R package [117], and defined the relative fitness of each population as *r*_*evo*_*—r*_*anc*_. Here, *r*_*evo*_ is the growth rate of the evolved population and *r*_*anc*_ is the mean growth rate of the three replicates of the ancestor grown in the same plate reported in units of cell divisions per hour. We measured each growth curve three times. We used the R package lme4 v1.1–12 [118] to perform a linear mixed effects analysis of the relationship between the evolved fitness relative to the ancestor and the mutation rate class. In this analysis, we chose the mutation rate classes as fixed effects, and the replicate population as well as the 96-well plate as random effects. For all linear mixed effects analyses conducted in this paper, we observed no deviations from homoscedasticity according to Levene's test for homogeneity of variance [119] implemented in the R package car v2.1–2 [120]. Also, all residuals were normally distributed unless otherwise specified. We obtained significance values using a likelihood ratio test of the full model against a null model that did not contain the fixed effects. Using the data from the above growth curve experiments, we also compared the fitness of the ancestor populations against each other by obtaining the relative fitness of the ancestors as *r*_*anc*_*—r*_*K12*_, where *r*_*anc*_ is the growth rate of the ancestor population and *r*_*K12*_ is the mean growth rate of the three replicates of *E*. *coli* MG1655 K12 grown in the same plate. We performed a linear mixed effects analysis of the relationship between the ancestral fitness relative to *E*. *coli* K12 and the mutation rate class using the lme4 package, as just described. In this analysis, we chose the mutation rate classes as fixed effects, and the identity of the original glycerol stock and of the 96-well plate as random effects. We used the R package multcomp v1.4–6 [121] to test whether fitness values had changed from the ancestral or reference state of 0.

### Sequencing

We sequenced samples from the four ancestral populations (day 0, generation 7) and from each of the 32 evolving replicate populations at days 63, 119, and 175 (generations 1046, 1977, and 2907). In total, we thus sequenced 100 populations (4 + 3×32). For simplicity, we hereafter designate these time points as generations 0, 1000, 2000, and 3000. For each, we isolated the DNA directly from cell pellets obtained from the evolving populations using Qiagen's DNeasy Blood and Tissue kit (cat. No 69582), with modifications as previously described [122]. We used the TruSeq DNA PCR-Free kit (Illumina FC-121-3002) to prepare and barcode the libraries for paired-end sequencing, as previously described [122]. Importantly, we used no PCR steps in preparing the libraries. We employed qPCR with Roche’s FastStart Essential DNA Green Master kit (Cat no. 06402712001) to quantify the libraries, which were then mixed in equimolar amounts for sequencing. We sequenced the populations (paired-end, 125 bp) on a single lane of Illumina’s HiSeq 2500 v2. We used *breseq* v0.26.1 [56] to align the reads, and call and annotate the variants relative to the *E*. *coli* K12 MG1655 reference genome NC_000913.3 [110], downloaded from the NCBI (https://www.ncbi.nlm.nih.gov/nuccore/556503834) on January 6, 2015. We developed scripts in R to identify the alterations that occurred in the evolved populations, but were not fixed in their ancestors. We determined mutational spectra by identifying all mutations that occurred at any detectable frequency in each population at every sequenced time point, and classified them into the following categories: A→C, A→G, A→T, C→A, C→G, or C→T. (Because DNA is double-stranded, the remaining possible point mutations are covered by their reverse complements, e.g., T→G corresponds to A→C.) We computed the relative frequencies of each mutational class for each replicate population, and used these to perform a principal component analysis (PCA) in R with prcomp, which uses singular value decomposition for the PCA. All data are available from the Dryad Digital Repository: https://doi.org/10.5061/dryad.mh206.

### Diversity and mutational cloud metrics

To quantify the movement and spread of a population as a “cloud” of sequences in sequence space, we first defined the center of this mutational cloud at any given site *n* in the genome as the majority allele *e*_*n*_*∈{A*,*C*,*G*,*T}*, i.e., the allele whose frequency *p* exceeded 0.5 (all sites we analyzed had one such allele). We defined the center of the mutational cloud of genomes as the location in genotype space defined by the majority allele at each site. It can also be viewed as the location of the population’s consensus sequence. If we denote the fraction of a population with the majority allele at site *n* as *p*, then the distance of the population to a given majority allele at site *n* can be thought of as the fraction of the population not having the majority allele, which is given as *C*_*n*_ *= (1—p)*. We define the population spread metric *C* as the average of *C*_*n*_ over all sites in the genome. A related quantity is the approximate sequence distance *D* that an evolving population has moved from its ancestral genotype, i.e.,

$$D=\sum_{n}\left\{ \begin{array}{c} \begin{array}{cc} 0, & \mathrm{if}\;a_{n}=e_{n} \end{array}\\ \begin{array}{cc} 1, & \mathrm{otherwise} \end{array} \end{array}\right.$$

where *a*_*n*_*∈{A*,*C*,*G*,*T}* is the ancestral allele of the population at generation 0. In other words, *D* corresponds to the total number of sites at which the majority allele is different from the ancestral allele. We also computed each population's average genome-scale nucleotide site diversity [123,124] using the pairwise alignment position nucleotide counting approach [125,126]. We estimated the proportion of pairwise nucleotide differences at each site *n* as

$$p_{n}=\frac{m_{p}(1-m_{p})}{m(m-1)/2},$$

where *m*_*p*_ is the number of reads corresponding to the majority allele and *m* is the total number of reads at site *n*. We estimated the average nucleotide diversity for the *L* positions in our genome having non-zero coverage as

$$\pi =\sum_{n=1}^{L}\frac{P_{n}}{L}.$$

We used the R package lme4 v1.1–12 [118] to perform a linear mixed effects analysis of the relationship between the cube root of *C* or *D* (taken to ensure homoscedasticy) and the mutation rate class. In this analysis, we chose the mutation rate classes as fixed effects, and the time points and each of the 32 evolving replicates as random effects. We obtained significance values using a likelihood ratio test of the full model against a null model that did not contain the fixed effects.

### Identification of putatively beneficial mutations

We identified putatively beneficial mutations as mutations that occurred in a genomic region more often than one would expect by chance alone. To identify such mutations, we used a numerical approach that focuses on a given gene *g* among a larger set of genes or genomic regions *G* (e.g., a gene among the set of all genes), and asked whether more replicate population experienced a high-frequency genetic change than expected by chance. To this end, we first counted the number *n*_*g*_ of replicate populations with a mutation in gene *g* that had reached a frequency greater than 50% at generation 3000.

If all sites in the genomes of all samples were equally likely to experience a mutation, and if different genes were likely to experience mutations only in proportion to their length, then the probability *p*_*g*_ that any one gene *g* receives such a mutation in any given replicate would depend only on the length of the gene *l*_*g*_,

$$p_{g}=\frac{l_{g}}{\sum_{\gamma \in G}l_{\gamma }}.$$

The *n*_*g*_ total mutations found in gene *g* could be distributed in (32ng) ways across the 32 replicate populations *R = {MR*^*S*^_*1*_,*…MR*^*S*^_*8*_,*MR*^*M*^_*1*_,*…MR*^*M*^_*8*_,*MR*^*L*^_*1*_,*…MR*^*L*^_*8*_,*MR*^*XL*^_*1*_,*…MR*^*XL*^_*8*_*}*. For example, for *n*_*g*_ = 2, the mutations could be distributed across the 32 replicate populations in (322) = 496 ways, i.e., one could occur in MR^S^_1_ and the other in MR^S^_2_, one could occur in MR^S^_1_ and the other in MR^S^_3_, etc. We computed the probability of observing the *n*_*g*_ mutations in any given set of replicates as the probability that gene *g* was mutated in each member of the set of replicates times the probability that it was not mutated in any of the other replicates. For *n*_*g*_ = 2 and replicate populations *r*_*i*_,*r*_*j*_ ∊*R*, this quantity is given by the binomial distribution adjusted to account for the number of observed mutant genes in the replicate populations, *n*_*ri*_ and *n*_*rj*_

$$p_{r_{i},r_{j}}=n_{r_{i}}n_{r_{j}}p_{g}^{n_{g}}{\left(1-p_{g}\right)}^{\left(n_{tot}-n_{r_{i}}-n_{r_{j}}\right)},$$

where

$$n_{tot}=\sum_{r\in R}n_{r}.$$

The probability of observing exactly *n*_*g*_ = 2 mutations in gene *g* in any pair of replicate populations is the sum of the probabilities that *n*_*g*_ mutations occurred in each of the 496 pairs, and is given by

$$P_{g}=\sum_{r_{i},r_{j}\in R,\;r_{i}\neq r_{j}}p_{r_{i},r_{j}}.$$

This quantity *P*_*g*_ is our null expectation that two replicates acquire mutations in gene *g*, if each replicate population's mutations were randomly distributed across its genome. We were interested in genes containing mutations in improbably many replicate populations, which we identified as those genes having less than a 0.005 percent chance of finding *n*_*g*_ replicate populations with a high frequency derived allele in the gene. We performed analogous analyses for *n*_*g*_ *> 2*. For example, for genes in which we observed three replicates with mutations (*n*_*g*_ *= 3*), we computed the probability that three replicates *r*_*i*_, *r*_*j*_, and *r*_*k*_ each contained a mutation in gene *g* as

$$p_{r_{i},r_{j},r_{k}}=n_{r_{i}}n_{r_{j}}n_{r_{k}}p_{g}^{n_{g}}{\left(1-p_{g}\right)}^{\left(n_{tot}-n_{r_{i}}-n_{r_{j}}-n_{r_{k}}\right)}.$$

Similarly, the probability of observing a mutation in exactly 3 replicate populations is given by

$$P_{g}=\sum_{r_{i},r_{j},r_{k}\in R,\;r_{i}\neq r_{j}\neq r_{k}}p_{r_{i},r_{j},r_{k}}.$$

### Mutation rate measurements and calculations

We estimated the mutation rate of a single clone isolated from each ancestor and from each evolved replicate population through fluctuation assays that screened for mutants resistant to rifampicin [127], which can be caused by mutations in the *rpoB* gene. Specifically, we performed the following procedure for each replicate population. We isolated a single random clone and incubated it overnight in 2 mL DM1000 in 48 well plates at 37°C with shaking. We diluted the resulting overnight culture 100,000-fold to yield a culture with approximately 1000 cells in 100 μL. We then transferred 100 μL of the diluted culture into 5–7 sterile 50 mL tubes (Sarstedt 62.547.254) containing 30 mL of DM1000, and incubated for 48 hours at 37°C with shaking. We estimated the number of cells in each tube by plating dilutions on LB agar plates, and estimated the number of resistant cells in each tube by plating dilutions on LB agar plates supplemented with 50 mg/mL rifampicin (Sigma R3501-5G). We calculated the mutation rate to rifampicin *μ*_*rif*_ using the method and program provided by Philip Gerrish [108]. We obtained the genomic mutation rate *U* using Drake's approach [74] by first determining the "correction factor" *C*, which counts the number of single nucleotide mutations in *rpoB* that show rifampicin resistance. By counting all possible nucleotide changes underlying the amino acid changes in *rpoB* previously shown to confer rifampicin resistance [57], we determined that C = 71. Finally, we estimated the genomic mutation rate as *U = Lμ*_*rif*_*/C*, and the mutation rate per base pair as *μ = μ*_*rif*_*/C*, where *L* = 4641652 is the number of nucleotides in the *E*. *coli* K12 genome. This mutation rate may be an underestimate because we neglected other types of mutations (e.g., indels) and mutations in other genes that may lead to rifampicin resistance.

### Phenotype screening

Our phenotype screening revolved around the density of cells after growth in various chemicals. Specifically, we determined the cell density after 24 hours of growth using Biolog Phenotype MicroArrays PM11C, PM12B, PM13B, and PM14A MicroPlates (Biolog, Inc., Hayward CA, USA), which assay the sensitivity of bacteria to diverse chemicals that range from antibiotics to heavy metals. We screened the ancestors at generation 0, and two randomly selected replicates of the evolved populations (MR^S^_1_, MR^S^_6_, MR^M^_3_, MR^M^_4_, MR^L^_2_, MR^L^_7_, MR^XL^_3_, MR^XL^_4_) at the final time point of laboratory evolution. To do so, we streaked population samples from glycerol stocks onto LB agar plates, incubated them at 37°C for 24 hours, restreaked the resulting colonies onto fresh LB agar plates (37°C, 24 hours), and repeated this streaking and incubation procedure once more. We resuspended the colonies from the final (third round) plates in IF-0 solution (Biolog, Inc., Hayward CA, USA) to a final absorbance reading at 600 nm of approximately 0.18 (200 μL suspension in a 96-well TPP plate, ref 92096). We diluted this suspension 6-fold using IF-0+dye (Biolog, Inc., Hayward CA, USA), and diluted the resulting suspension 201-fold using IF-10+dye (Biolog, Inc., Hayward CA, USA). We added 100 μL of the final solution to each well of a Phenotype MicroArray and incubated the array in the dark at 37°C for 24 hours, taking absorbance readings at 600 nm after 10 minutes and 24 hours. We then computed the Biolog phenotype *B*_*S*,*C* =_ *A*_*S*,*C*,*24h*_*−A*_*S*,*C*,*10m*_, where *A*_*S*,*C*,*24h*_ and *A*_*S*,*C*,*10m*_ are the absorbance readings for each sample *S* and compound *C* at 600 nm after 24 hours and 10 minutes.

To determine the minimum threshold for detection of growth in a given compound *C*, we computed the absolute difference between the readings in a given well across all pairs of samples *(i*,*j)* after 10 minutes (before cells had started to grow and divide), i.e., *A*_*i-j*,*C*,*10m*_ *= |A*_*i*,*C*,*10m*_*−A*_*j*,*C*,*10m*_*|*. The values of *A*_*i-j*,*C*,*10m*_ quantify the expected experimental noise of wells with no growth. We found that *A*_*i-j*,*C*,*10m*_<0.097 for more than 99% of sample pairs. Based on this observation, we considered differences between readings smaller than the threshold value A_thresh_ = 0.097 as due to experimental noise.

Each compound in the Biolog Phenotype MicroArrays we used occurs in four wells at increasing concentrations. For further analysis, we used data only from the concentration (the well) that showed the highest variation in the difference between matched evolved and ancestor strains across all samples. We considered a sample to have evolved tolerance to a compound *C* if it improved its phenotype after 3000 generations of evolution more than expected based on experimental noise, i.e., *B*_*SEvo*,*C*_ *− B*_*SAnc*,*C*_ *> A*_*thresh*_. Likewise, we considered that a sample had lost tolerance if its phenotype had degenerated after 3000 generations of evolution, i.e., if *B*_*SAnc*,*C*_ *− B*_*SEvo*,*C*_ *> A*_*thresh*_. We note that both cellular growth and respiration contribute to the Biolog phenotype *B*_*S*,*C*_, because respiration can occur independently of cellular growth [70,128].

We were also interested in observing the evolutionary dynamics of phenotypes over time. The phenotypes we selected for this analysis are the cell density after 24 hours of growth of the evolved populations relative to their ancestors in two conditions: a narrow antibiotic (nitrofurantoin) stress, and a broader environmental (low pH) stress. Specifically, we chose DM1000 medium with 1.5–2.4 μg/mL nitrofurantoin for the narrow antibiotic stress, and acidic DM1000 (pH 4–5.25) for the broad stress. Nitrofurantoin is one of the phenotypes where evolved populations gained tolerance in the Biolog analyses, and acid stress has been well-studied in *E*. *coli* [72]. To control for changes in cell density at stationary phase, we also performed a control measurement in the standard medium, DM1000. Specifically, we measured the growth of evolved replicate populations at days 28, 63, 91, 119, 147, and 175 (generations 465, 1046, 1511, 1977, 2442, and 2907, hereafter designated as generations 500, 1000, 1500, 2000, 2500, and 3000). To do so, we inoculated each population in triplicate from glycerol stock in DM1000 and grew it at 37°C for at least 18 hours. We then diluted the resulting culture 50-fold into DM1000 medium immediately before adding 10 μL from the diluted culture to 190 μL media with nitrofurantoin, low pH, or just DM1000 in a 96-well plate. We incubated the resulting 96-well plates for 24 hours, and then measured the absorbance at 600 nm. We computed the normalized fold change in cell density in each condition at six time points by obtaining the average value of *G = (A*_*X*,*E*_ */ A*_*X*,*A*_*) / (A*_*DM1000*,*E*_ */ A*_*DM1000*,*A*_*)* for the three replicate cultures, where *A*_*X*,*Y*_ is the absorbance reading in condition *X* (e.g., 2.2 μg/mL nitrofurantoin or acidic DM1000 medium) of a given replicate before evolution (*Y = A* for ancestral) or after evolution (*Y = E* for evolved). The denominator, *(A*_*DM1000*, *E*_ */ A*_*DM1000*, *A*_*)*, removes the effect of changes in the evolved carrying capacity, which otherwise could confound cell density changes observed in stressful media with evolved cell density changes in the medium without stressor. We considered that evolution had increased cell density relative to the ancestor when the numerator of G was greater than its denominator, *(A*_*X*, *E*_ */ A*_*X*, *A*_*) > (A*_*DM1000*, *E*_ */ A*_*DM1000*, *A*_). In order to quantify the relationship between the normalized fold-change in cell density *G* and ancestral mutation rate, we performed a linear mixed effects analysis using the R package lme4 v1.1–12 [118] to obtain the relationship between *G* and the mutation rate class. In this analysis, we chose the mutation rate classes (MR^S^, MR^M^, MR^L^, and MR^XL^) as fixed effects, and the measurement time points, the experimental condition, and each of the 32 evolving replicates as random effects. For the nitrofurantoin and pH stressors, we used data from the experimental condition with the most variability between replicates (2.3 μg/mL nitrofurantoin and pH 5.25) in this analysis. We tested for homoscedasticity using the R package car v 2.1–2 [120], and found that the untransformed pH data and the log-transformed nitrofurantoin data were homoscedastic (Levene's test, pH 5.25: F_3,188_ = 1.71, p = 0.17; nitrofurantoin 2.3 μg/mL: F_3,188_ = 1.25, p = 0.29).

## Supporting information

S1 Fig Cell density (vertical axes) after 24 hours of growth as a function of generation time (horizontal axes). We counted the number of cells in stationary phase just before our daily transfer at regular intervals. Each point is the average cell density of an evolving replicate population at a given generation. One standard deviation above and below the mean is depicted with a shaded line. Different colors distinguish data from the MR^S^ (blue), MR^M^ (yellow), MR^L^ (orange), and MR^XL^ (red) strains.
(PDF)

S2 Fig Effective population size (N_e_) of replicate populations over the course of the evolution experiment. We counted the number of cells at regular intervals, and used these counts to estimate (A) the nominal effective population size N_e_ for each replicate population. Because our populations are asexual, the effects of selection on polymorphisms linked to neutral sites will make drift at neutral sites appear much stronger than indicated by these estimates. To account for such effects, we also made rough estimates of the effect of linkage on the effective population size using two published methods (further described in Methods), which compute the "Gordo" N_e_ (B), and the "Good" N_e_ (C). Together, panels B and C suggest that the effective population size may be much smaller than the nominal population size. Each circle shows the N_e_ estimate of a replicate population, the center line of the box plot is the median value, and the top and bottom edges of the box correspond to the first and third quartiles. Different colors distinguish data from the MR^S^ (blue), MR^M^ (yellow), MR^L^ (orange), and MR^XL^ (red) strains.
(PDF)

S3 Fig (A) Fitness differences between ancestral replicate populations and *E*. *coli* K12 MG1655. Each circle shows the growth rate of a replicate population for a given strain (horizontal axis) minus the growth rate of *E*. *coli* K12 MG1655 from the same experimental batch. Overall, 54 experimental estimates were made for each strain. (B) Fitness differences between each evolving replicate population and a common reference strain *E*. *coli* K12 MG1655 over time are depicted in separate panels for each strain and replicate. Shaded areas indicate one s.e.m. Different colors distinguish data from the MR^S^ (blue), MR^M^ (yellow), MR^L^ (orange), and MR^XL^ (red) strains.
(PDF)

S4 Fig (A) (C) The fitness difference between each evolving replicate population and its ancestor and its change over time is depicted in separate panels for each strain and replicate. Panels corresponding to the replicates randomly chosen for further characterization in Biolog plates are outlined with a heavy black border. Shaded areas indicate one s.e.m. (B) Variance in relative fitness for the replicate populations of each strain. Strains with higher ancestral mutation rates have more variability in the relative fitness of their evolving populations than those with lower mutation rates. Different colors distinguish data from the MR^S^ (blue), MR^M^ (yellow), MR^L^ (orange), and MR^XL^ (red) strains.
(PDF)

S5 Fig Percentage of the genome with no sequencing coverage for all 100 sequenced populations. Different colors distinguish data from the MR^S^ (blue), MR^M^ (yellow), MR^L^ (orange), and MR^XL^ (red) strains.
(PDF)

S6 Fig Frequency and type of SNP in each evolving population over time. Each line in a given panel shows the frequency of one SNP in one replicate population (vertical axis) at generations 0, 1000, 2000, and 3000 (horizontal axis). The color of the line indicates the type of SNP. Types of SNPs with likely functional consequences are emphasized in brown (nonsense mutations) and green (nonsynonymous mutations). Data from all eight independently-evolving replicates (rows of panels) are plotted for each strain (MR^S^, MR^M^, MR^L^, and MR^XL^; columns of panels).
(PDF)

S7 Fig The frequency of newly-arising SNPs after one day of growth in the ancestral populations. Several of the observed SNPs, particularly those occurring at higher frequencies, may have been transferred to the eight replicates. Different colors distinguish data from the MR^S^ (blue), MR^M^ (yellow), MR^L^ (orange), and MR^XL^ (red) strains.
(PDF)

S8 Fig The mutational spectra at four-fold degenerate sites for each evolving replicate strain. (A) Nucleotide changes are depicted along the horizontal axis. For each type of mutation, we computed how often it occurred at any time point during the evolution experiment relative to all other types (Methods). (B) The mutational spectra from replicate populations evolved from ancestors with different mutation rates do not clearly separate when projected onto the first two principal components (PC1 and PC2) in a principal component analysis (Methods). (C) The scree plot shows that PC1 and PC2 account for 43% and 32% of the variability, respectively. Different colors distinguish data from the MR^S^ (blue), MR^M^ (yellow), MR^L^ (orange), and MR^XL^ (red) strains.
(PDF)

S9 Fig The number of replicates for which at least half of the population harbors any mutation in a putatively beneficial gene. Each dot indicates that a single replicate acquired a mutation within the gene and that this mutation rose to a frequency of at least 50% in a given generation (horizontal axes). In this analysis, we included all genes (rows, labeled in left-most subpanel of each row) that rose to a frequency of >50% in significantly more replicate populations at generation 3000 than expected by chance alone (see Methods). Because we evolved eight replicate populations for each strain, each vertical stack of dots can harbor at most eight dots. For many genes, all MR^XL^ replicates share the same nucleotide change, which likely already occurred in the shared ancestor. (A) Genes with different mutations in the same gene in different replicates, and (B) genes where all the MR^XL^ replicates share the same nucleotide change (the nucleotide changes found in the MR^L^ replicate populations for *betI* and *torA* are not the same as found in the MR^XL^ populations). Different colors distinguish data from the MR^S^ (blue), MR^M^ (yellow), MR^L^ (orange), and MR^XL^ (red) strains.
(PDF)

S10 Fig The evolutionary dynamics of mutations in the eight putatively beneficial genes. Each circle corresponds to one evolving replicate population. The size of a circle is proportional to the frequency at which a mutation is found in a population, and can change over time (horizontal axes). All replicates for all strains (circles inside each panel) are depicted for each gene (labeled in the top, left of each panel). Different colors distinguish data from the MR^S^ (blue), MR^M^ (yellow), MR^L^ (orange), and MR^XL^ (red) strains.
(PDF)

S11 Fig Cell density of two randomly selected evolved and ancestral strains in 96 different environments on Biolog plates. Importantly, all tested ancestor and evolved MR^XL^ strains failed to grow in every one of the 96 environments. Each circle represents the ancestor's density (horizontal axes) and the evolved replicate population's density (vertical axes) in a particular environment. Points above the diagonal line correspond to conditions in which an evolved replicate population outperformed its ancestor; points below the line correspond to conditions in which an evolved replicate population underperformed its ancestor. We consider a population to have evolved tolerance to a condition when its density is larger than the ancestral density in the same condition, excluding differences attributable to experimental noise. Conversely, we consider a population as having experienced decay if its density after evolution is smaller than that of its ancestor (see Methods). Both gains and decays are indicated by solid circles. Open circles indicate that no gain or decay was detected for that condition, or that the difference between the evolved and ancestral cell density could be due to experimental noise. Different colors distinguish data from the MR^S^ (blue), MR^M^ (yellow), and MR^L^ (orange) strains.
(PDF)

S12 Fig The fold-change in cell density after 24 hours of growth of evolved replicate populations (relative to their ancestor) in media with nitrofurantoin and low pH media. We measured the cell density of the MR^S^, MR^M^, MR^L^, and MR^XL^ evolved replicate populations relative to their ancestors over the course of the experiment (horizontal axis) in (A) the antibiotic nitrofurantoin (1.5 μg/mL—2.4 μg/mL), and (B) acidic media (pH 4.0–5.25). Different colors distinguish data from the MR^S^ (blue), MR^M^ (yellow), MR^L^ (orange), and MR^XL^ (red) strains.
(PDF)

S13 Fig Comparisons of multiple population properties (matrix diagonal) for the experimental data from each evolved replicate population. We plotted each property in a pairwise fashion to identify correlations between properties. Each property is listed on the diagonal ("log(U)" is the logarithm of the genomic mutation rate, "relative fitness" is the evolved growth rate relative to the ancestor, "N_e_" is the effective population size, "cell density at 3000" is the absorbance reading at 600 nm at generation 3000 after 24 hours of growth in minimal medium, "log(derived alleles)" is the logarithm of the number of high frequency derived alleles at generation 3000, "log(cloud size)" is the logarithm of the population's average distance to the center of the cloud at generation 3000, "log(pH cell density)" is the logarithm of the normalized fold change in cell density after 24 hours of growth in acidic media at pH 5.25, and "log(nitro cell density)" is the logarithm of the normalized fold change in cell density after 24 hours of growth in media containing 2.2 μg/mL nitrofurantoin). Pairwise comparisons are plotted below the diagonal; each circle corresponds to a different replicate population. The Spearman correlation coefficient of each panel below the diagonal is reported in the corresponding panel above the diagonal. Different colors distinguish data from the MR^S^ (blue), MR^M^ (yellow), MR^L^ (orange), and MR^XL^ (red) strains.
(PDF)

S14 Fig No evidence that the mutation rate genome is preferentially subject to genetic change. (A) We calculated the percentage of synonymous nucleotide changes (at any frequency) that occurred within genes belonging to the mutation rate genome (vertical axes) during the evolution experiment (horizontal axes) at any frequency in each evolving replicate population (circles). Horizontal gray lines indicate the percentage of coding regions in the E. coli genome that belong to the mutation rate genome (2.8%). There are no more mutations in the mutation rate genome than expected by chance alone at generation 3000 (one-sided binomial test, MR^S^:n = 55,p = 1.0; MR^M^:n = 48,p = 1.0; MR^L^:n = 433,p = 0.79; MR^XL^:n = 1050,p = 0.99). (B) We calculated the mean synonymous nucleotide site diversity and its standard error (Methods). The mean synonymous nucleotide site diversity for the mutation rate genome is depicted in the right panel, and for all other genes in the left panel. Note that no or very few sites may contribute to average diversity at low mutation rates. Shaded areas indicate one standard error of the mean. Different colors distinguish data from the MR^S^ (blue), MR^M^ (yellow), MR^L^ (orange), and MR^XL^ (red) strains.
(PDF)

S15 Fig The evolutionary dynamics of possibly function-altering mutations in the mutation rate genome. Each circle corresponds to a putatively function-altering mutation (nonsynonymous or nonsense mutations in protein-coding genes, or any mutation in tRNA-encoding genes) in one evolving replicate population. The size of a circle is proportional to the frequency at which a mutation is found in a population, and can change over time (horizontal axes). All replicates for all strains (circles inside each panel) are depicted for each gene (labeled on the top left of each panel). Different colors distinguish data from the MR^S^ (blue), MR^M^ (yellow), MR^L^ (orange), and MR^XL^ (red) strains.
(PDF)

S1 Table All single nucleotide differences between the ancestor strains MR^S^, MR^M^, MR^L^, and MR^XL^. The columns are as follows: "Position" is the genomic coordinate of the SNP; "Ref" is the consensus ancestral sequence; "MR^S^", "MR^M^", "MR^L^", and " MR^XL^ " display the SNPs in the each of the respective ancestral strains (a period means that the nucleotide is the same as in the consensus ancestral sequence); "Gene" is the name of the gene in or near which the SNP occurs; "SNP type" defines what effect the polymorphism may have (e.g., nonsynonymous, intergenic, noncoding); and "AA effect" is a SNP's effect on the protein sequence for SNPs occurring within protein-coding genes. SNPs occurring in intergenic regions are annotated with the nearest 5' and 3' genes.
(XLSX)

S2 Table Genes putatively involved in modulating the mutation rate. (XLSX)

S3 Table Genomic mutation rates. The columns are as follows: "Strain" is the identity of the ancestral strain (e.g., MR^S^, MR^M^, MR^L^, MR^XL^); "Replicate" identifies the replicate population; "μ" is the mutation rate in units of number of mutations per bp per cell generation; "μ 95% confidence intervals" "Upper" and "Lower" give the confidence in the mutation rate estimate; "*U*" is the genomic mutation rate in number of mutations per genome and per cell generation; "*U* 95% confidence intervals" "Upper" and "Lower" give the confidence in the genomic mutation rate estimate; "*N*_*e*_*U*" is the estimated number of mutations to occur in an evolving population each generation. Mutation rates were measured at generations 0 ("Ancestor" replicates) and 3000 (all other replicates).
(XLSX)

S1 Text Area Under the Curve (AUC) is a complementary fitness metric that also demonstrates reduced adaptation at very high mutation rates. (PDF)

S2 Text Applicability of several theoretical models predicting loss or reduction of adaptation at high mutation rates. (PDF)

S3 Text The waiting time for the establishment of a new beneficial allele. (PDF)
