# Supplementary figures and images for: High mutation rates limit evolutionary adaptation in Escherichia coli
Source: PLoS Genet. 2018 Apr 27;14(4):e1007324. doi: 10.1371/journal.pgen.1007324 (PMC5942850; doi:10.1371/journal.pgen.1007324)

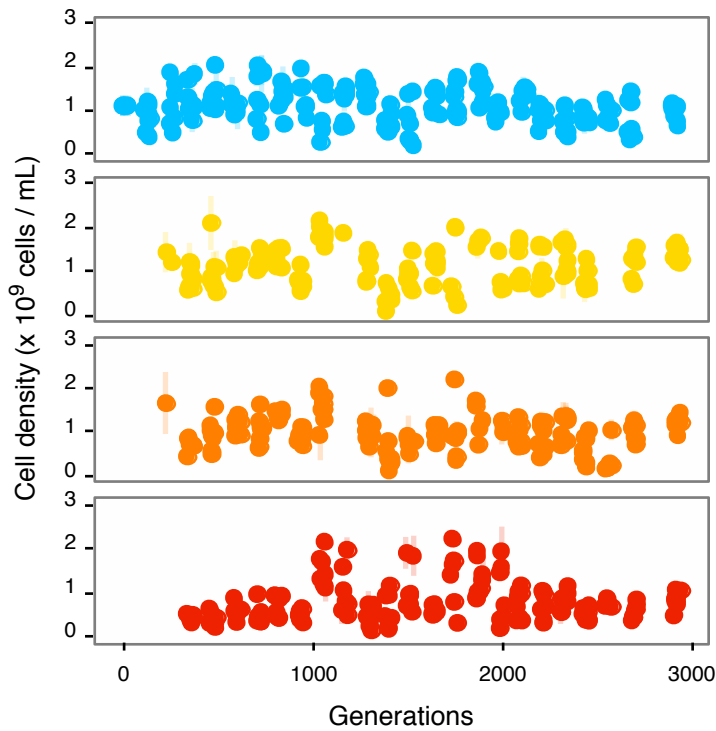

Supplement: S1 Fig — We counted the number of cells in stationary phase just before our daily transfer at regular intervals. Each point is the average cell density of an evolving replicate population at a given generation. One standard deviation above and below the mean is depicted with a shaded line. Different colors distinguish data from the MRS (blue), MRM (yellow), MRL (orange), and MRXL (red) strains. (PDF) [file pgen.1007324.s001.pdf]

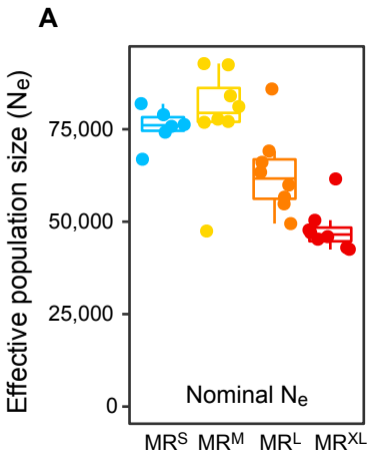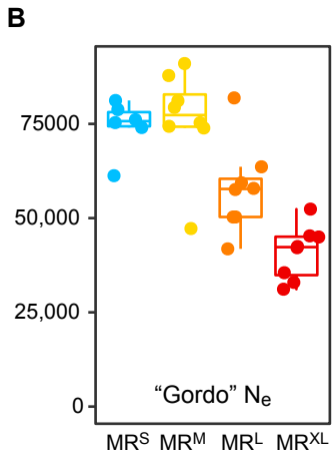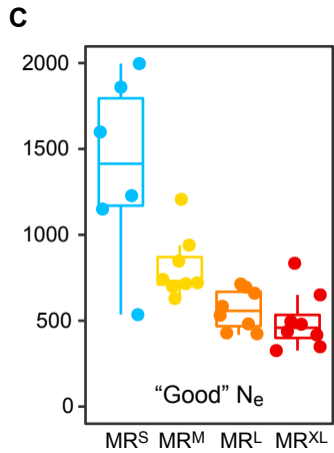

Supplement: S2 Fig — We counted the number of cells at regular intervals, and used these counts to estimate (A) the nominal effective population size Ne for each replicate population. Because our populations are asexual, the effects of selection on polymorphisms linked to neutral sites will make drift at neutral sites appear much stronger than indicated by these estimates. To account for such effects, we also made rough estimates of the effect of linkage on the effective population size using two published methods (further described in Methods), which compute the "Gordo" Ne (B), and the "Good" Ne (C). Together, panels B and C suggest that the effective population size may be much smaller than the nominal population size. Each circle shows the Ne estimate of a replicate population, the center line of the box plot is the median value, and the top and bottom edges of the box correspond to the first and third quartiles. Different colors distinguish data from the MRS (blue), MRM (yellow), MRL (orange), and MRXL (red) strains. (PDF) [file pgen.1007324.s002.pdf]

**A**

Ancestor's fitness difference  
from *E. coli* K12

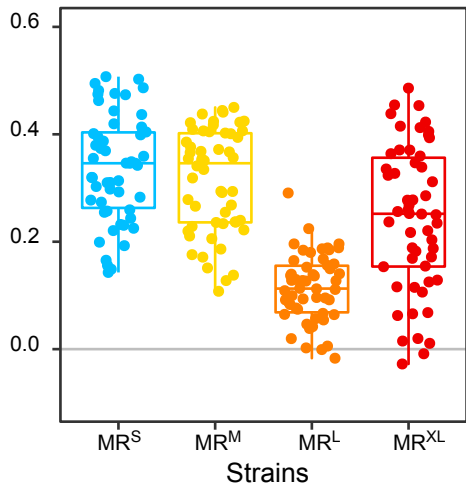**B**

Fitness difference from *E. coli* K12

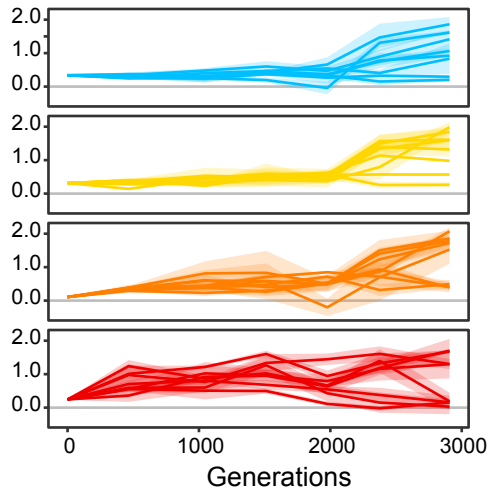

Supplement: S3 Fig — (A) Fitness differences between ancestral replicate populations and E. coli K12 MG1655. Each circle shows the growth rate of a replicate population for a given strain (horizontal axis) minus the growth rate of E. coli K12 MG1655 from the same experimental batch. Overall, 54 experimental estimates were made for each strain. (B) Fitness differences between each evolving replicate population and a common reference strain E. coli K12 MG1655 over time are depicted in separate panels for each strain and replicate. Shaded areas indicate one s.e.m. Different colors distinguish data from the MRS (blue), MRM (yellow), MRL (orange), and MRXL (red) strains. (PDF) [file pgen.1007324.s003.pdf]

**A**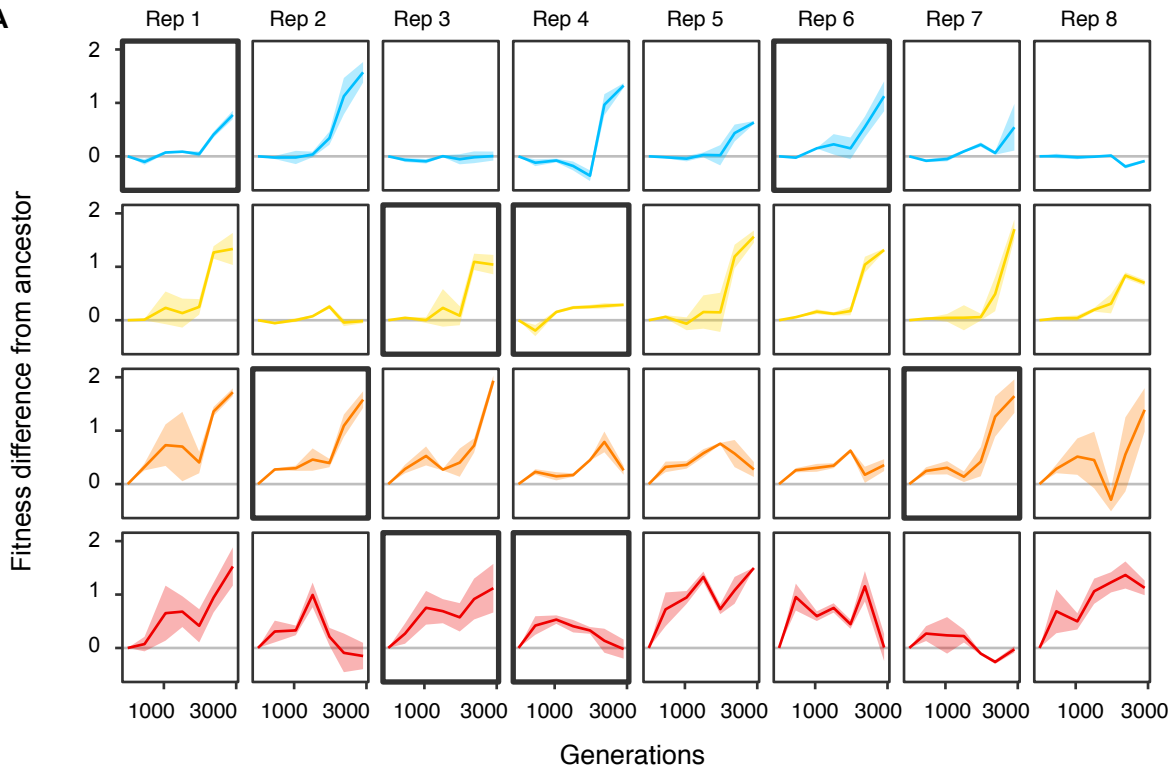**B**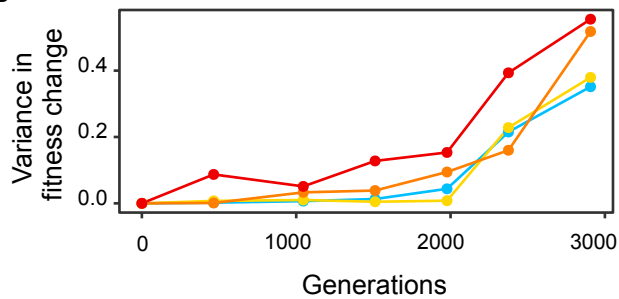

Supplement: S4 Fig — (A) (C) The fitness difference between each evolving replicate population and its ancestor and its change over time is depicted in separate panels for each strain and replicate. Panels corresponding to the replicates randomly chosen for further characterization in Biolog plates are outlined with a heavy black border. Shaded areas indicate one s.e.m. (B) Variance in relative fitness for the replicate populations of each strain. Strains with higher ancestral mutation rates have more variability in the relative fitness of their evolving populations than those with lower mutation rates. Different colors distinguish data from the MRS (blue), MRM (yellow), MRL (orange), and MRXL (red) strains. (PDF) [file pgen.1007324.s004.pdf]

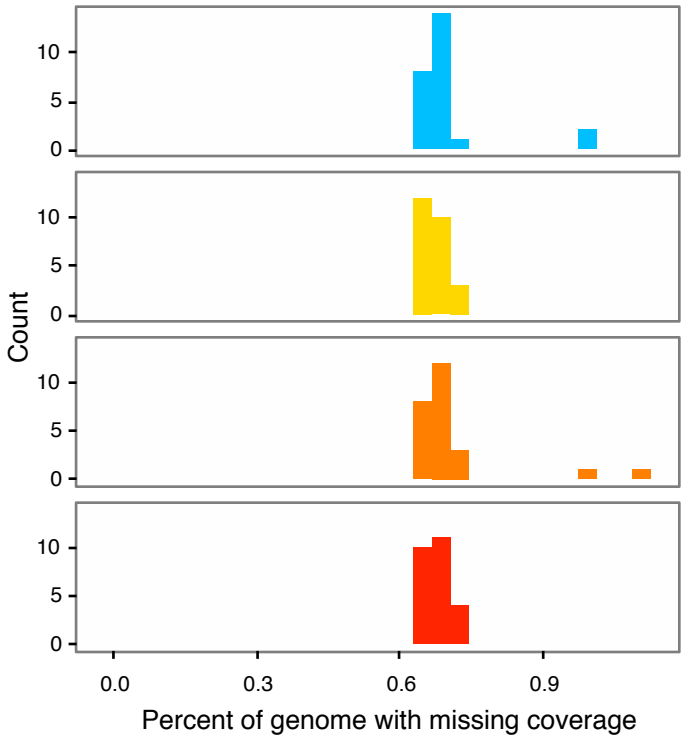

Supplement: S5 Fig — Different colors distinguish data from the MRS (blue), MRM (yellow), MRL (orange), and MRXL (red) strains. (PDF) [file pgen.1007324.s005.pdf]

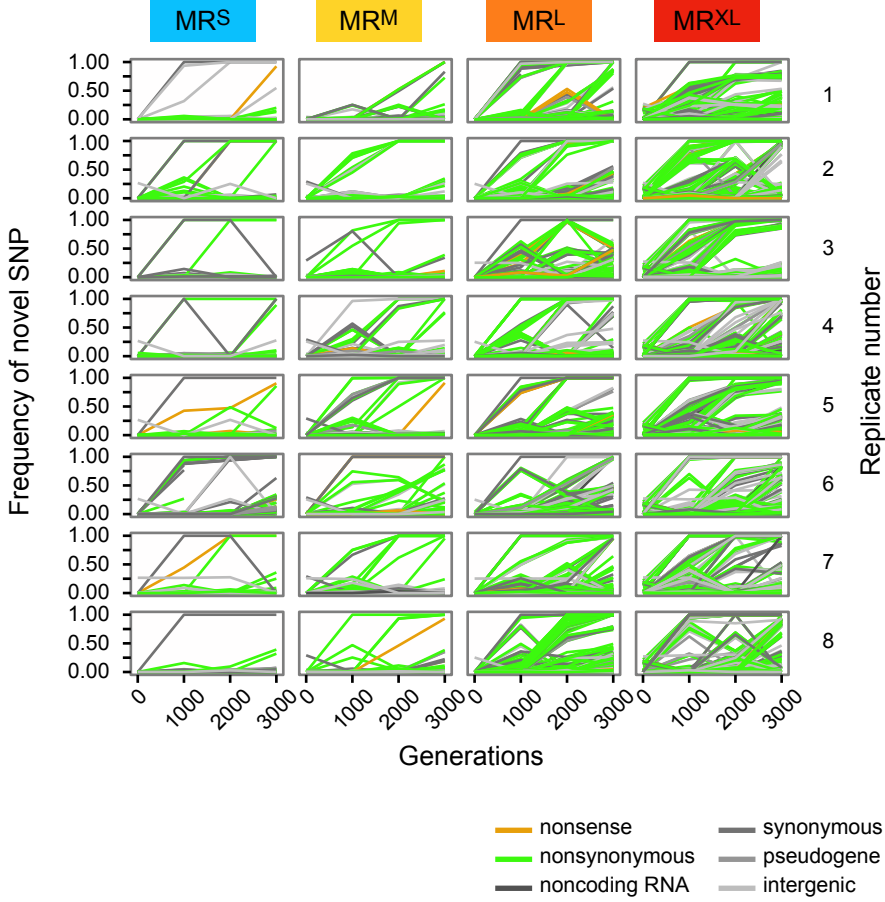

Supplement: S6 Fig — Each line in a given panel shows the frequency of one SNP in one replicate population (vertical axis) at generations 0, 1000, 2000, and 3000 (horizontal axis). The color of the line indicates the type of SNP. Types of SNPs with likely functional consequences are emphasized in brown (nonsense mutations) and green (nonsynonymous mutations). Data from all eight independently-evolving replicates (rows of panels) are plotted for each strain (MRS, MRM, MRL, and MRXL; columns of panels). (PDF) [file pgen.1007324.s006.pdf]

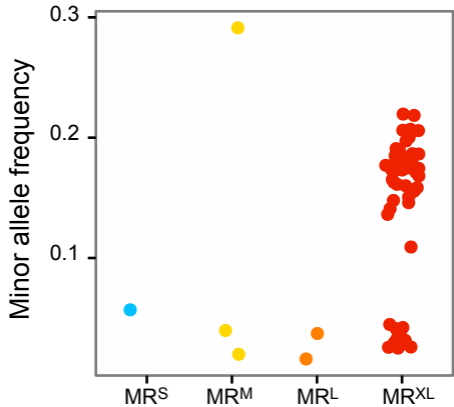

Supplement: S7 Fig — The frequency of newly-arising SNPs after one day of growth in the ancestral populations. Several of the observed SNPs, particularly those occurring at higher frequencies, may have been transferred to the eight replicates. Different colors distinguish data from the MRS (blue), MRM (yellow), MRL (orange), and MRXL (red) strains. (PDF) [file pgen.1007324.s007.pdf]

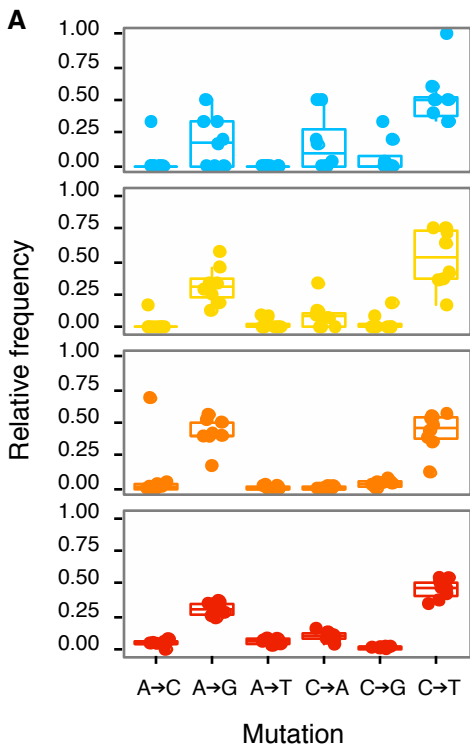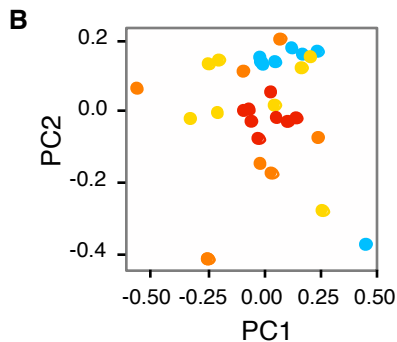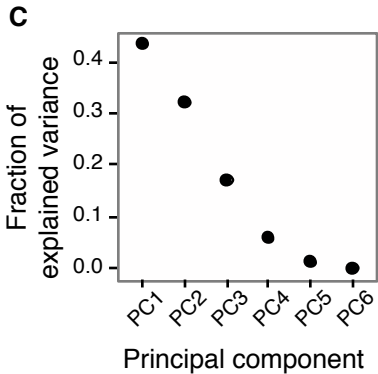

Supplement: S8 Fig — (A) Nucleotide changes are depicted along the horizontal axis. For each type of mutation, we computed how often it occurred at any time point during the evolution experiment relative to all other types (Methods). (B) The mutational spectra from replicate populations evolved from ancestors with different mutation rates do not clearly separate when projected onto the first two principal components (PC1 and PC2) in a principal component analysis (Methods). (C) The scree plot shows that PC1 and PC2 account for 43% and 32% of the variability, respectively. Different colors distinguish data from the MRS (blue), MRM (yellow), MRL (orange), and MRXL (red) strains. (PDF) [file pgen.1007324.s008.pdf]

**A**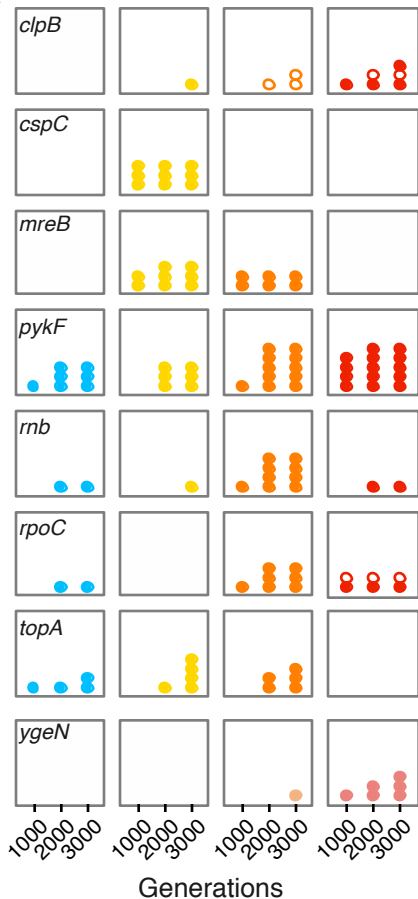

- nonsynonymous, nonsense
- synonymous
- pseudogene
- noncoding

**B**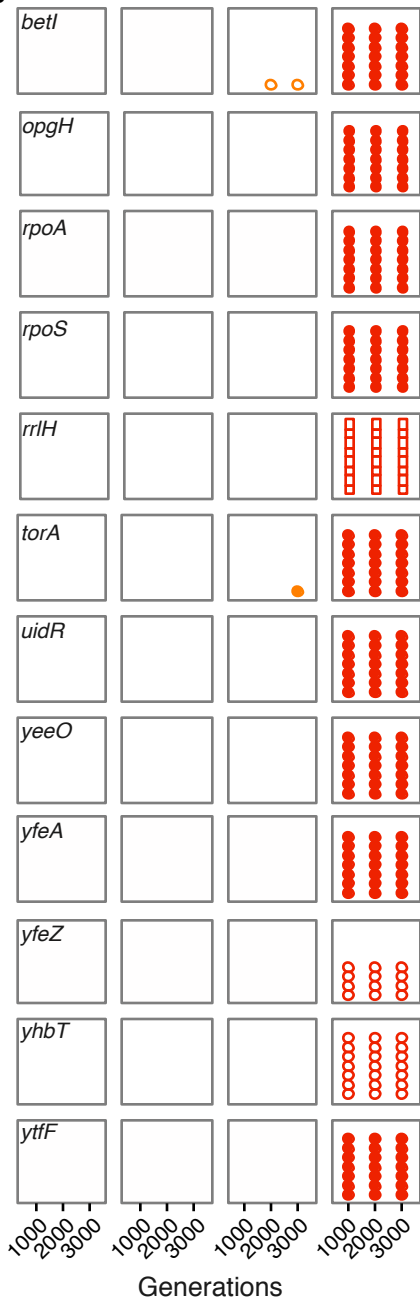

Supplement: S9 Fig — Each dot indicates that a single replicate acquired a mutation within the gene and that this mutation rose to a frequency of at least 50% in a given generation (horizontal axes). In this analysis, we included all genes (rows, labeled in left-most subpanel of each row) that rose to a frequency of >50% in significantly more replicate populations at generation 3000 than expected by chance alone (see Methods). Because we evolved eight replicate populations for each strain, each vertical stack of dots can harbor at most eight dots. For many genes, all MRXL replicates share the same nucleotide change, which likely already occurred in the shared ancestor. (A) Genes with different mutations in the same gene in different replicates, and (B) genes where all the MRXL replicates share the same nucleotide change (the nucleotide changes found in the MRL replicate populations for betI and torA are not the same as found in the MRXL populations). Different colors distinguish data from the MRS (blue), MRM (yellow), MRL (orange), and MRXL (red) strains. (PDF) [file pgen.1007324.s009.pdf]

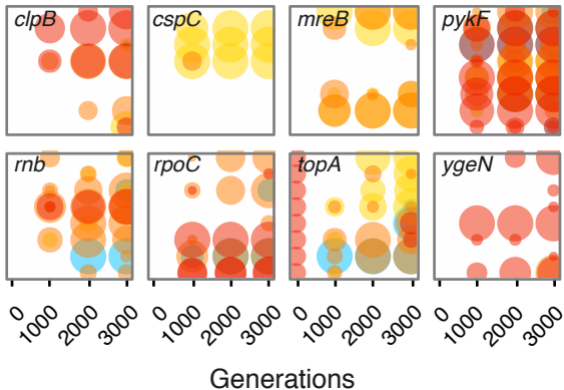

Supplement: S10 Fig — Each circle corresponds to one evolving replicate population. The size of a circle is proportional to the frequency at which a mutation is found in a population, and can change over time (horizontal axes). All replicates for all strains (circles inside each panel) are depicted for each gene (labeled in the top, left of each panel). Different colors distinguish data from the MRS (blue), MRM (yellow), MRL (orange), and MRXL (red) strains. (PDF) [file pgen.1007324.s010.pdf]

Evolved cell density

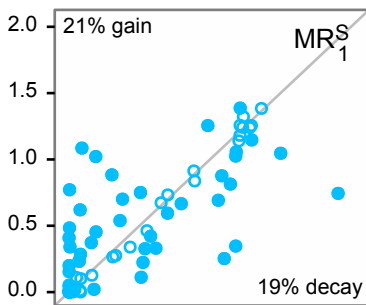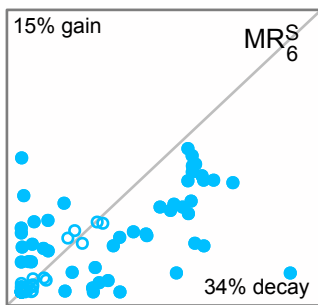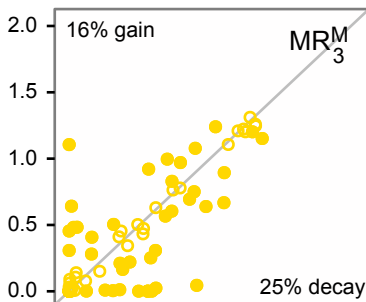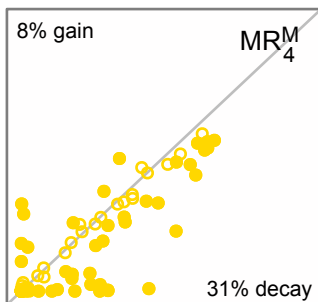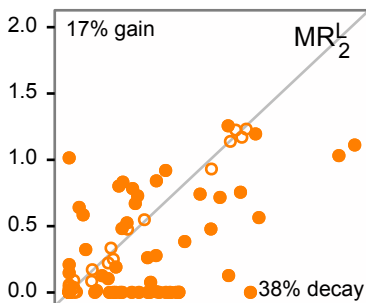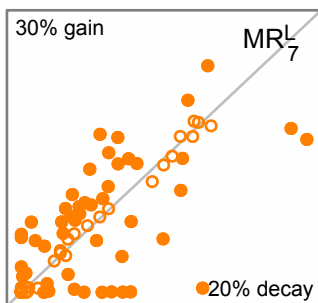

Ancestor cell density

Supplement: S11 Fig — Importantly, all tested ancestor and evolved MRXL strains failed to grow in every one of the 96 environments. Each circle represents the ancestor's density (horizontal axes) and the evolved replicate population's density (vertical axes) in a particular environment. Points above the diagonal line correspond to conditions in which an evolved replicate population outperformed its ancestor; points below the line correspond to conditions in which an evolved replicate population underperformed its ancestor. We consider a population to have evolved tolerance to a condition when its density is larger than the ancestral density in the same condition, excluding differences attributable to experimental noise. Conversely, we consider a population as having experienced decay if its density after evolution is smaller than that of its ancestor (see Methods). Both gains and decays are indicated by solid circles. Open circles indicate that no gain or decay was detected for that condition, or that the difference between the evolved and ancestral cell density could be due to experimental noise. Different colors distinguish data from the MRS (blue), MRM (yellow), and MRL (orange) strains. (PDF) [file pgen.1007324.s011.pdf]

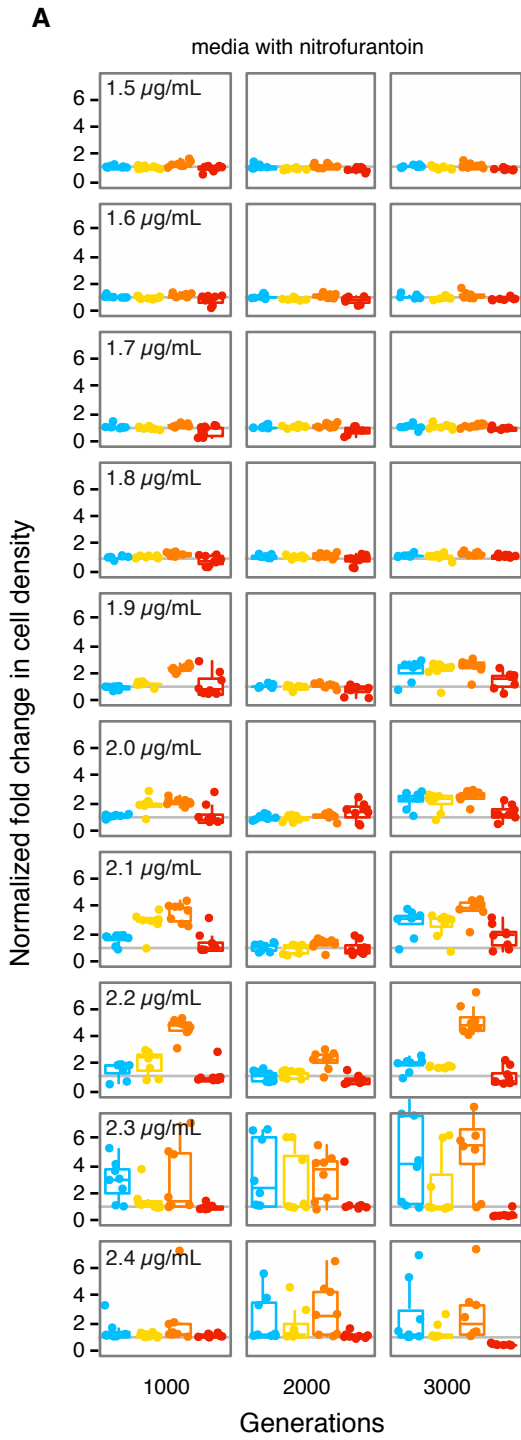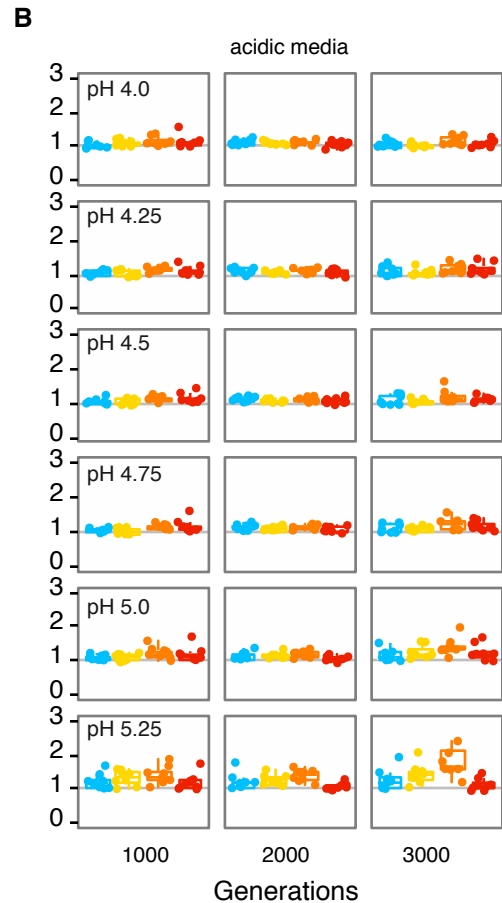

Supplement: S12 Fig — We measured the cell density of the MRS, MRM, MRL, and MRXL evolved replicate populations relative to their ancestors over the course of the experiment (horizontal axis) in (A) the antibiotic nitrofurantoin (1.5 μg/mL—2.4 μg/mL), and (B) acidic media (pH 4.0–5.25). Different colors distinguish data from the MRS (blue), MRM (yellow), MRL (orange), and MRXL (red) strains. (PDF) [file pgen.1007324.s012.pdf]

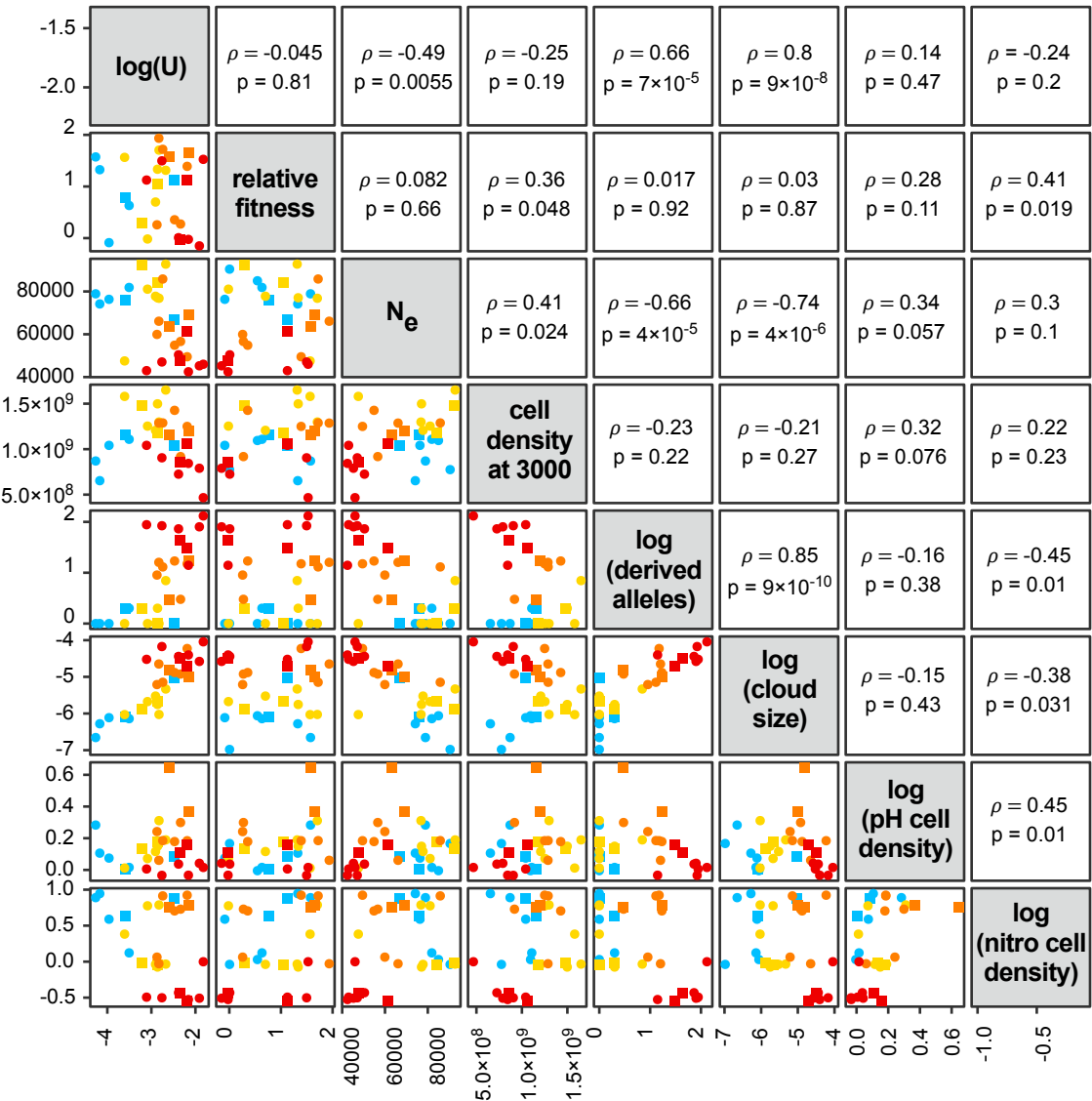

Supplement: S13 Fig — We plotted each property in a pairwise fashion to identify correlations between properties. Each property is listed on the diagonal ("log(U)" is the logarithm of the genomic mutation rate, "relative fitness" is the evolved growth rate relative to the ancestor, "Ne" is the effective population size, "cell density at 3000" is the absorbance reading at 600 nm at generation 3000 after 24 hours of growth in minimal medium, "log(derived alleles)" is the logarithm of the number of high frequency derived alleles at generation 3000, "log(cloud size)" is the logarithm of the population's average distance to the center of the cloud at generation 3000, "log(pH cell density)" is the logarithm of the normalized fold change in cell density after 24 hours of growth in acidic media at pH 5.25, and "log(nitro cell density)" is the logarithm of the normalized fold change in cell density after 24 hours of growth in media containing 2.2 μg/mL nitrofurantoin). Pairwise comparisons are plotted below the diagonal; each circle corresponds to a different replicate population. The Spearman correlation coefficient of each panel below the diagonal is reported in the corresponding panel above the diagonal. Different colors distinguish data from the MRS (blue), MRM (yellow), MRL (orange), and MRXL (red) strains. (PDF) [file pgen.1007324.s013.pdf]

**A**

Percent of mutation rate genome  
with synonymous mutations

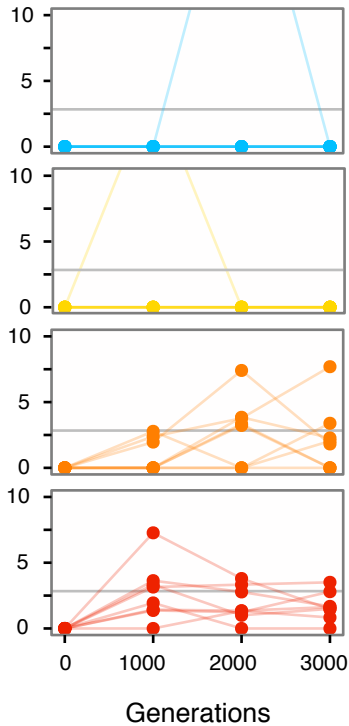**B**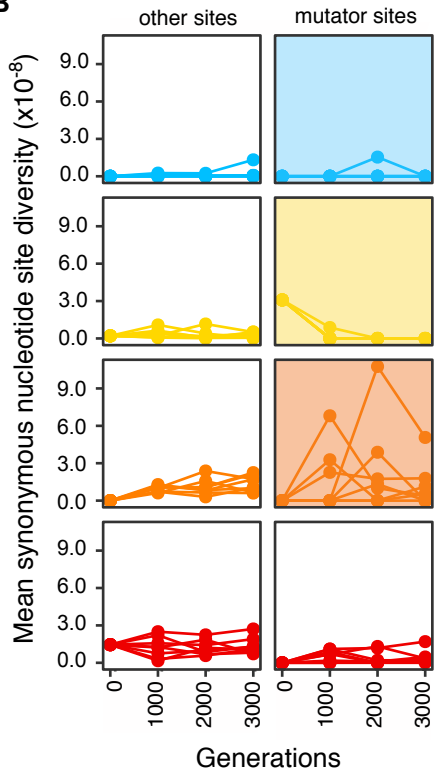

Supplement: S14 Fig — (A) We calculated the percentage of synonymous nucleotide changes (at any frequency) that occurred within genes belonging to the mutation rate genome (vertical axes) during the evolution experiment (horizontal axes) at any frequency in each evolving replicate population (circles). Horizontal gray lines indicate the percentage of coding regions in the E. coli genome that belong to the mutation rate genome (2.8%). There are no more mutations in the mutation rate genome than expected by chance alone at generation 3000 (one-sided binomial test, MRS:n = 55,p = 1.0; MRM:n = 48,p = 1.0; MRL:n = 433,p = 0.79; MRXL:n = 1050,p = 0.99). (B) We calculated the mean synonymous nucleotide site diversity and its standard error (Methods). The mean synonymous nucleotide site diversity for the mutation rate genome is depicted in the right panel, and for all other genes in the left panel. Note that no or very few sites may contribute to average diversity at low mutation rates. Shaded areas indicate one standard error of the mean. Different colors distinguish data from the MRS (blue), MRM (yellow), MRL (orange), and MRXL (red) strains. (PDF) [file pgen.1007324.s014.pdf]

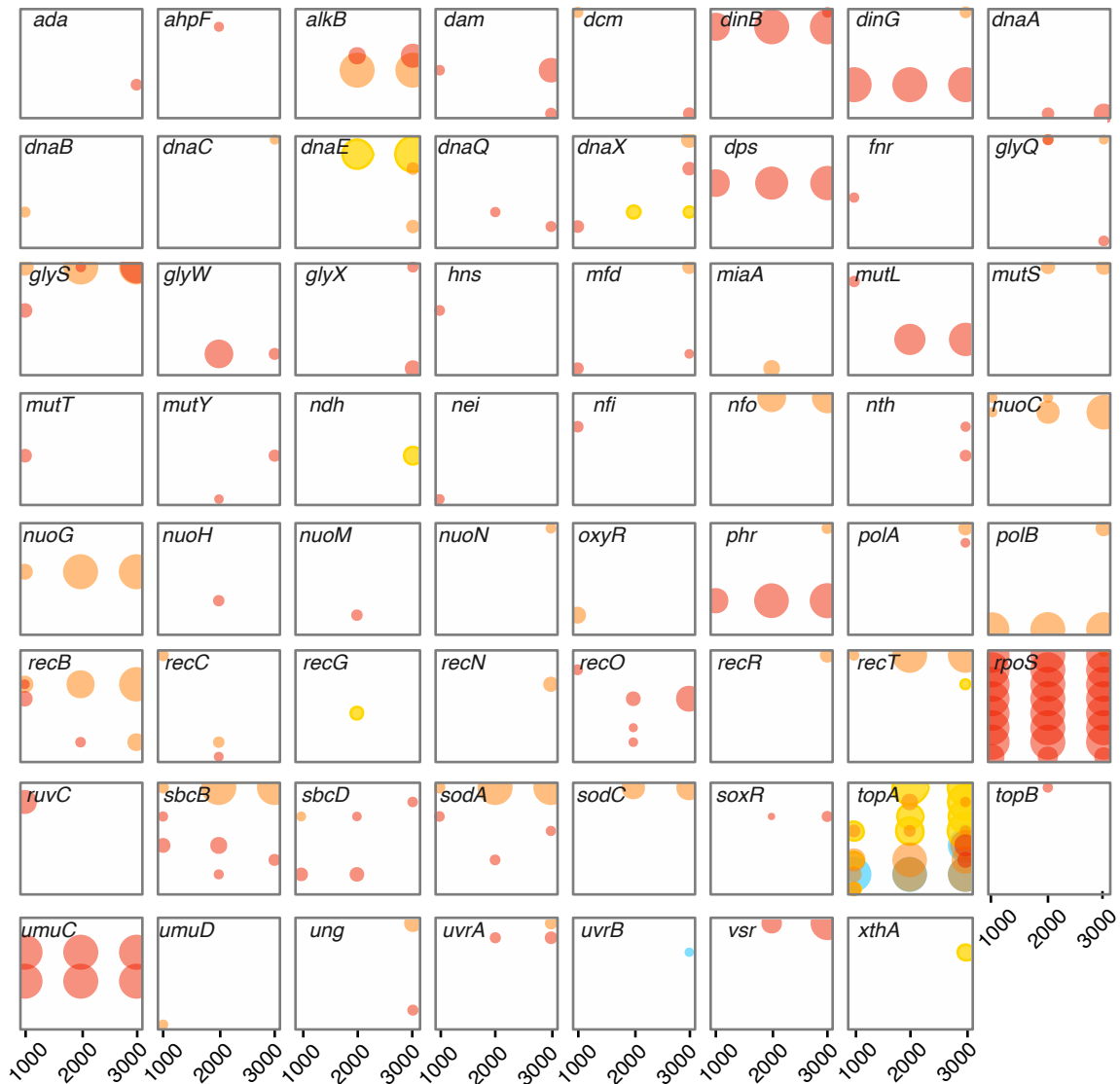

Generations

Supplement: S15 Fig — Each circle corresponds to a putatively function-altering mutation (nonsynonymous or nonsense mutations in protein-coding genes, or any mutation in tRNA-encoding genes) in one evolving replicate population. The size of a circle is proportional to the frequency at which a mutation is found in a population, and can change over time (horizontal axes). All replicates for all strains (circles inside each panel) are depicted for each gene (labeled on the top left of each panel). Different colors distinguish data from the MRS (blue), MRM (yellow), MRL (orange), and MRXL (red) strains. (PDF) [file pgen.1007324.s015.pdf]
