# Supplementary material for: High mutation rates limit evolutionary adaptation in Escherichia coli
Source: PLoS Genet. 2018 Apr 27;14(4):e1007324. doi: 10.1371/journal.pgen.1007324 (PMC5942850; doi:10.1371/journal.pgen.1007324)
Supplement: S1 Text — (PDF) [file pgen.1007324.s019.pdf]

Text S1. Area Under the Curve (AUC) is a complementary fitness metric that also demonstrates reduced adaptation at very high mutation rates.

The growth curve metric Area Under the Curve (AUC) integrates information about lag phase, growth rate, and carrying capacity by summing up the area under the growth curve. A form of this metric that can be obtained without the need to fit models of population growth is known as the empirical AUC (eAUC) [117]. By integrating information from the entire growth curve, the eAUC metric alleviates an important limitation of using maximum growth rate as a sole fitness proxy. To find out whether this more comprehensive fitness measure fundamentally alters our observations on population fitness, we here used the difference between the evolved and ancestral empirical AUC as a fitness measure (Fig. A), rather than the maximum growth rate as in the main text. Our observations based on maximum growth rate remain unchanged. Strains with higher mutation rates still show a greater increase in fitness, except for the MR^{XL} strain, which had the smallest fitness increase of all strains (MR^S: 1.1±0.3; MR^M: 0.98±0.08; MR^L: 1.22±0.08; MR^{XL}: 0.3±0.1, mean relative fitness ± s.e.m.).

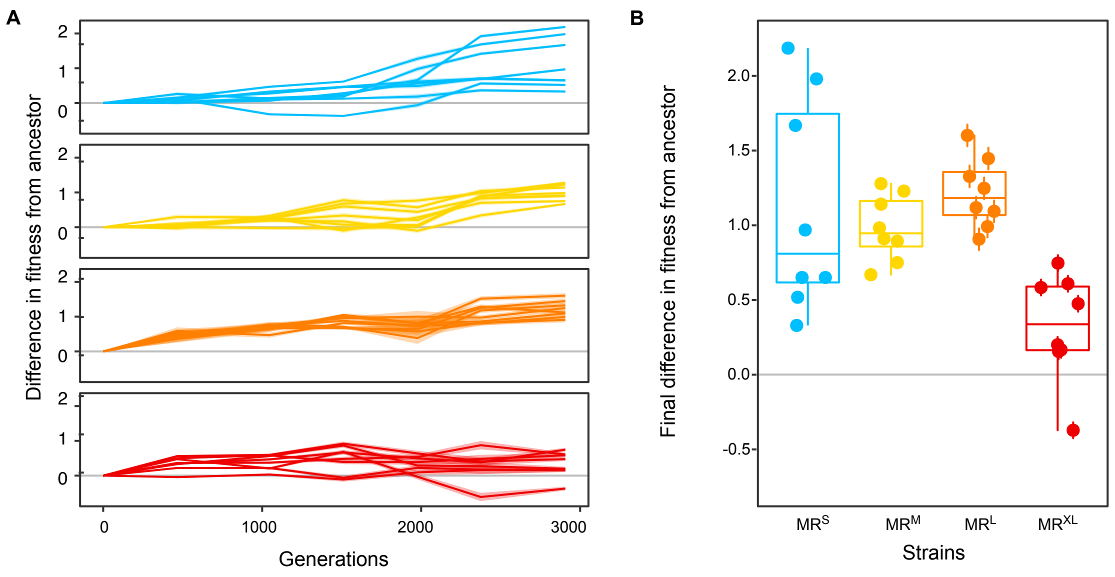

Fig A. The fitness proxy AUC of evolving replicate populations relative to their ancestors (A) over time, and (B) at the end of the experiment. A relative fitness value greater than zero indicates that the evolved population has higher fitness than its ancestor. Different colors distinguish data from the MR^S (blue), MR^M (yellow), MR^L (orange), and MR^{XL} (red) populations. Shaded areas (A) or bars (B) indicate standard errors.
