# Supplementary material for: High mutation rates limit evolutionary adaptation in Escherichia coli
Source: PLoS Genet. 2018 Apr 27;14(4):e1007324. doi: 10.1371/journal.pgen.1007324 (PMC5942850; doi:10.1371/journal.pgen.1007324)
Supplement: S2 Text — (PDF) [file pgen.1007324.s020.pdf]

Text S2. Applicability of several theoretical models predicting loss or reduction of adaptation at high mutation rates.

We observed that MR^{XL} replicate populations, on average, no longer show a fitness gain. Further, we found that three of the eight replicates show a fitness loss. We wanted to find out whether existing theory could explain such reduced adaptation and fitness loss. To this end, we examined multiple models that aim to explain the effects of mutation on adaptation, including models pertaining to lethal mutagenesis [20], Eigen's error threshold [19,23,129], Muller's Ratchet [92,93], mutational meltdown [22], and genetic load. When choosing parameters for these models, we aimed to select conservative values, that is, values which would result in the most extreme predicted effects on fitness. For example, we sometimes used the total genomic mutation rate when a model required the deleterious genomic mutation rate, or reduced the estimated effective population size to account for stronger drift at neutral sites in linkage disequilibrium with selective alleles in our asexual strains. Despite such conservative assumptions, the models we discuss below cannot explain the reduced adaptation and fitness loss we observe at high mutation rates. We also note that these models below make some unrealistic assumptions about our experimental populations. These include (but are not limited to) assumptions about the distribution of fitness effects of new mutations, and the extent to which our populations are in equilibrium. We nonetheless discuss these models, because they illustrate that prominent existing models cannot explain the experimental phenomena we observe.

Lethal mutagenesis

Lethal mutagenesis is an approach to kill pathogen populations by artificially raising their mutation rates to increase the deleterious genetic load [20,100,130], which is predicted to occur in bacteria when the deleterious genomic rate U_d exceeds *0.69* [20]. The maximum observed genomic mutation rate U observed in our experiments was found in the MR^{XL} ancestor ($U = 0.036$), far below the threshold for lethal mutagenesis. Thus, our populations are unlikely to lose fitness through lethal mutagenesis.

Error threshold

The concept of the error threshold is based on models describing asexually reproducing populations called quasi-species. It is defined as the mutation rate above which a genome can no longer maintain the information encoded in it [129]. Populations (quasi-species) evolving with mutation rates above the error threshold are expected to spread across sequence space and eventually lose their genetic structure (and thus viability). Fitness in quasispecies models can be described by classical populations genetics. Indeed, equations describing the fitness of quasi-species can be equivalent to those describing mutation-selection equilibrium, and a population's reduction in average fitness at equilibrium can be determined by a population's mutational load (described in [131]). We discuss our populations' mutation load and its expected effect on fitness further in the section below entitled "Genetic Load."

Muller's Ratchet

Muller's Ratchet refers to the accumulation of deleterious alleles in a finite asexually reproducing (non-recombining) population, which can lead to declining absolute fitness in the absence of beneficial mutations [92,93]. Models studying Muller's ratchet focus on the clone with the fewest deleterious mutations. All other clones in the population have more deleterious mutations and thus lower fitness, which means they will be eventually be eliminated by selection. The number of individuals in the clone with the fewest deleterious mutations is given by $N_0 = Ne^{-U_d/s}$ [93], where N is the population size, U_d is the genomic deleterious mutation rate, and s is the effect size of a deleterious mutation. Muller's Ratchet is likely to be important for a population when $N_e s e^{-U_d/s}$ is less than 15 [132]. Even when this condition holds, the loss of fitness by Muller's Ratchet can be counteracted by the presence of beneficial mutations [133] or recombination [134]. (Beneficial mutations may occur in our populations, but recombination is not likely to occur.) We estimated the values $N_e s\ e^{-U_d/s}$ for all of our MR^{XL} strains. The effects of selection at linked sites are likely to mean that drift at neutral site is likely to be much stronger than indicated by these estimates. To this end, we chose to also use values for the effective population size N_e that would provide conservatively small estimates of $N_e s\ e^{-U_d/s}$ by correcting for linkage according to two methods (as described in the Methods and [53]), and a value of $s = 0.03$ in accordance with published estimates [115]. We used U as an estimate of U_d, which is an upper bound for U_d and thus renders our estimates of $N_e s\ e^{-U_d/s}$ conservatively small. With these parameters, no samples fall below the $N_e s\ e^{-U_d/s} < 15$ threshold when we used nominal or background selection corrected estimates of N_e. However, we find that twelve samples dip below the threshold of $N_e s\ e^{-U_d/s} < 15$ when using the N_e corrected for increased interference in asexual populations (Fig B, diamonds; 1 MR^S, 5 MR^L, and 6 MR^{XL} replicate populations). The ratchet may be acting in this situation, but its effects could also be counteracted by beneficial or back mutations. In sum, despite our conservative choices of parameters, Muller's Ratchet is not likely to have a strong effect on our populations.

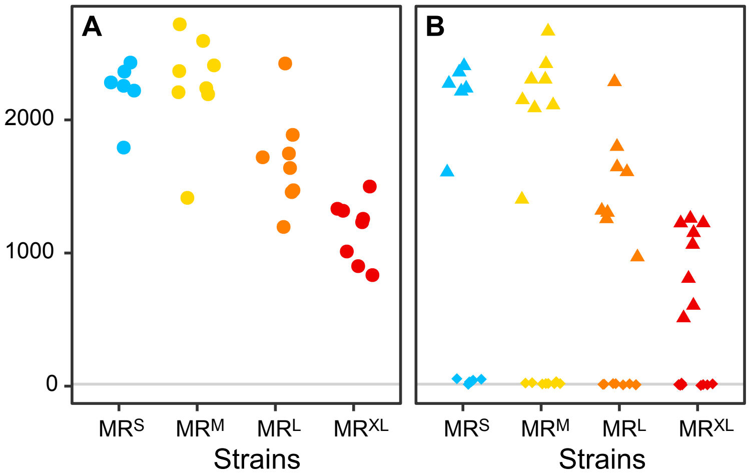

Fig B. Muller's Ratchet may be important for populations with values of $N_e s\ e^{-U_d/s}$ smaller than 15 (gray horizontal lines). (A) No samples fell below the threshold when we used the nominal effective population size for this analysis. (B) We also used two conservative estimates of N_e in our calculations to estimate the effects of linkage (diamonds and triangles, interference-corrected "Good N_e" and background-corrected "Gordo N_e", described in the Methods [53,55,132]). Twelve interference-corrected samples

(diamonds) drop below the threshold (one MR^S, five MR^L, and six MR^{XL} replicate populations). Different colors distinguish data from the MR^S (blue), MR^M (yellow), MR^L (orange), and MR^{XL} (red) strains.

Mutational Meltdown

Mutational meltdown models [22] can be thought of as incorporating demography into the process of Muller's Ratchet. Muller's Ratchet models consider fitness relative to those individuals in a population with the highest fitness (the so-called least loaded class), and do not define at what point in the ratcheting process a population is expected to go extinct. Mutational meltdown, on the other hand, explicitly considers the absolute fitness of a population, and predicts the extinction of populations whose fitness has declined so much that it cannot replace dying individuals with newborn individuals (mean viability is $1 / R$, where R is the number of surviving offspring per individual). Further, populations undergoing mutational meltdown are expected to shrink as their absolute fitness decreases (which in turn increases the likelihood that deleterious alleles persist in a population because of genetic drift). Populations subject to mutational meltdown would begin to irreversibly acquire deleterious mutations under the same conditions as for Muller's Ratchet (see previous section; Fig B). Once absolute fitness declines below the replacement rate, mutational meltdown and eventual extinction may occur. However, as we discussed in the previous section, the influence of Muller's Ratchet is not likely to be strong in our populations, and the prospect of mutational meltdown is thus even smaller, at least on the time scale of our experiment.

Genetic Load

Genetic load is the reduction in fitness of a population from the deleterious alleles found in a population at mutation-selection balance. Formally, $L = (w_{max} - w_{mean}) / w_{max}$, where w_{max} is the maximum fitness clone in a population and w_{mean} is the mean fitness of the population. For haploid, asexual populations, the genetic load is given as $L = 1 - e^{-Ud}$ [94]. We generated a conservative (i.e., possibly too high) estimate of the genetic load by using U as an upper bound for U_d (Fig C). We found the reduction in population fitness at equilibrium due to genetic load for our evolved MR^{XL} populations to be at most $L = 0.015$ ($MR^{XL}{}_1$), and on average $L = 0.006 \pm 0.002$ (MR^{XL} replicates, mean ± s.e.m.). In comparison, our estimates of the average genetic load of evolved replicates from the MR^L strain were $L = 0.004 \pm 0.001$ (MR^L replicates, mean ± s.e.m.). We expect the reduction in fitness due to genetic load in our evolved MR^{XL} populations to be relatively modest and not much greater than that found in strains with lower mutation rates — at most 1.5% and on average 0.6% (vs. 0.4% in MR^L). Thus, genetic load is not likely to have a strong effect on our populations.

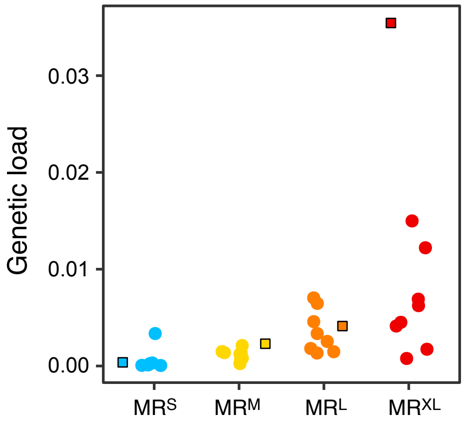

Fig C. Genetic load for the ancestral populations (squares) and evolved replicate populations (circles). A population's genetic load depends only on its deleterious mutation rate; the evolved MR^{XL} replicates have lower mutation rates (and thus carry less genetic load) than their ancestor. Different colors distinguish data from the MR^{S} (blue), MR^{M} (yellow), MR^{L} (orange), and MR^{XL} (red) strains.

Text S2 References

129. Eigen M. Selforganization of matter and the evolution of biological macromolecules. Naturwissenschaften. 1971;58: 465–523.

130. Bull JJ, Sanjuán R, Wilke CO. Theory of lethal mutagenesis for viruses. J Virol. 2007;81: 2930–2939. doi:10.1128/JVI.01624-06

131. Wilke CO. Quasispecies theory in the context of population genetics. BMC Evol Biol. 2005;5: 44. doi:10.1186/1471-2148-5-44

132. Gordo I, Charlesworth B. The speed of Muller's ratchet with background selection, and the degeneration of Y chromosomes. Genet Res. 2001;78: 149–161.

133. Bachtrog D, Gordo I. Adaptive evolution of asexual populations under Muller's ratchet. Evol Int J Org Evol. 2004;58: 1403–1413.

134. Gordo I, Campos PRA. Sex and deleterious mutations. Genetics. 2008;179: 621–626. doi:10.1534/genetics.108.086637
