# Supplementary material for: High mutation rates limit evolutionary adaptation in Escherichia coli
Source: PLoS Genet. 2018 Apr 27;14(4):e1007324. doi: 10.1371/journal.pgen.1007324 (PMC5942850; doi:10.1371/journal.pgen.1007324)
Supplement: S3 Text — (PDF) [file pgen.1007324.s021.pdf]

Text S3. The waiting time for the establishment of a new beneficial allele.

We estimated the waiting time for the establishment of a new beneficial allele as the inverse of the supply rate of beneficial mutations that survive drift ($1/(2Nus)$), using the nominal effective population size N_e for N, the mean effect size of a beneficial mutation $s_b = 0.086$ [135], and the beneficial mutation rate estimated as the expected fraction of the total genomic mutation rate that confers a beneficial mutation ($0.031 = u_b / u = 7.6\times 10^{-11}/2.47\times 10^{-9}$, from [135]), given by $U_b = 0.031U$. The longest expected wait to establish a beneficial mutation for any replication population is 44 generations (MR^{S}_{2}: $U = 5.4\times 10^{-5}$, $N_e = 78{,}900$).

Text S3 References

135. Kassen R, Bataillon T. Distribution of fitness effects among beneficial mutations before selection in experimental populations of bacteria. Nat Genet. 2006;38: 484–488. doi:10.1038/ng1751
